# *Megalictis*, the Bone-Crushing Giant Mustelid (Carnivora, Mustelidae, Oligobuninae) from the Early Miocene of North America

**DOI:** 10.1371/journal.pone.0152430

**Published:** 2016-04-07

**Authors:** Alberto Valenciano, Jon A. Baskin, Juan Abella, Alejandro Pérez-Ramos, M. Ángeles Álvarez-Sierra, Jorge Morales, Adam Hartstone-Rose

**Affiliations:** 1 Departamento de Geología Sedimentaria y Cambio Medioambiental, Instituto de Geociencias (CSIC, UCM), Madrid, Spain; 2 Departamento de Paleontología UCM, Facultad de Ciencias Geológicas UCM, Madrid, Spain; 3 Department of Biological and Health Sciences, Texas A&M University-Kingsville, Kingsville, United States of America; 4 Universidad Estatal Península de Santa Elena, La Libertad, Santa Elena, Ecuador; 5 Institut Català de Paleontologia Miquel Crusafont, Universitat Autònoma de Barcelona, Edifici ICP, Campus de la UAB, Cerdanyola del Vallès, Barcelona, Spain; 6 Departamento de Ecología y Geología, Facultad de Ciencias, Universidad de Málaga, Málaga, Spain; 7 Departamento de Paleobiología. Museo Nacional de Ciencias Naturales-CSIC, Madrid, Spain; 8 Department of Cell Biology and Anatomy, University of South Carolina School of Medicine, Columbia, South Carolina, United States of America; 9 Department of Anthropology, University of South Carolina, Columbia, South Carolina, United States of America; New York Institute of Technology College of Osteopathic Medicine, UNITED STATES

## Abstract

We describe cranial and mandibular remains of three undescribed individuals of the giant mustelid *Megalictis ferox* Matthew, 1907 from the latest Arikareean (Ar4), Early Miocene mammal fauna of Nebraska, and Wyoming (USA) housed at the American Museum of Natural History (New York, USA). Our phylogenetic hypothesis indicates that Ar4 specimens assigned to *M*. *ferox* constitute a monophyletic group. We assign three additional species previously referred to *Paroligobunis* to *Megalictis*: *M*. *simplicidens*, *M*. *frazieri*, and “*M*.” *petersoni*. The node containing these four species of *Megalictis* and *Oligobunis* forms the Oligobuninae. We test the hypothesis that Oligobuninae (*Megalictis* and *Oligobunis*) is a stem mustelid taxon. Our results indicate that the Oligobuninae form the sister clade to the crown extant mustelids. Based on the cranium, *M*. *ferox* is a jaguar-size mustelid and the largest terrestrial mustelid known to have existed. This new material also sheds light on a new ecomorphological interpretation of *M*. *ferox* as a bone-crushing durophage (similar to hyenas), rather than a cat-like hypercarnivore, as had been previously described. The relative large size of *M*. *ferox*, together with a stout rostrum and mandible made it one of the more powerful predators of the Early Miocene of the Great Plains of North America.

## Introduction

*Megalictis ferox* Matthew, 1907 [1] is a giant mustelid of the subfamily Oligobuninae and belongs to the paraphyletic group of “paleomustelids” [2]. It lived in the Early Miocene during the late Arikareean Ar4 North American Land Mammal Age 22.7–18.5 mya [3, 4] of the central Great Plains of United States in the states of Nebraska, South Dakota, and Wyoming [1, 5–7]. The Ar4 lithostratigraphic units containing giant oligobunines have been revised. Hunt [8] named the Anderson Ranch Formation for the terminal formation of the Arikaree Group in Nebraska and Wyoming formerly referred to as the Upper Harrison beds of Peterson [5, 9] and the lower Marsland Formation of Schultz [10]. The Black Bear Formation replaces the upper Rosebud Formation of South Dakota [11].

*Megalictis ferox* [1] was described from the Black Bear Formation, Stanley County, South Dakota, USA. A second giant oligobunine, *Aelurocyon brevifacies* Peterson, 1907 [5], was described from the Niobrara Canyon Local Fauna, Anderson Ranch Formation in Sioux County, Nebraska, USA. Hunt and Skolnick [7] established that the actual publication date for *A*. *brevifacies* was one week after Matthew described *M*. *ferox* in 1907, not in 1906 as indicated in the journal. After these initial descriptions, Riggs [6] described new cranial and postcranial material of both taxa. Hunt and Skolnick [7] synonymized *Megalictis ferox*, *Aelurocyon brevifacies*, and the large oligobunine mustelid *Paroligobunis simplicidens* (Peterson, 1907) [5].

Here, we describe an important unpublished sample of craniomandibular remains of *Megalictis ferox* (F:AM 25430, F:AM 54079, and AMNH 54076), housed at the American Museum of Natural History (New York, USA). Although F:AM 25430 and F:AM 54079 were found in the late 1930s and have been used to obtain metric, morpho-functional and phylogenetic data (e.g., [2, 7, 12–16]), they have never been fully described. Therefore, the main objective of the present paper is to describe these unpublished skulls and mandibles, and provide new data on the taxonomy and systematics of the genus in order to shed new light on the paleobiology of *Megalictis*.

## Material and Methods

### Nomenclature and Measurements

**Fig 1:**
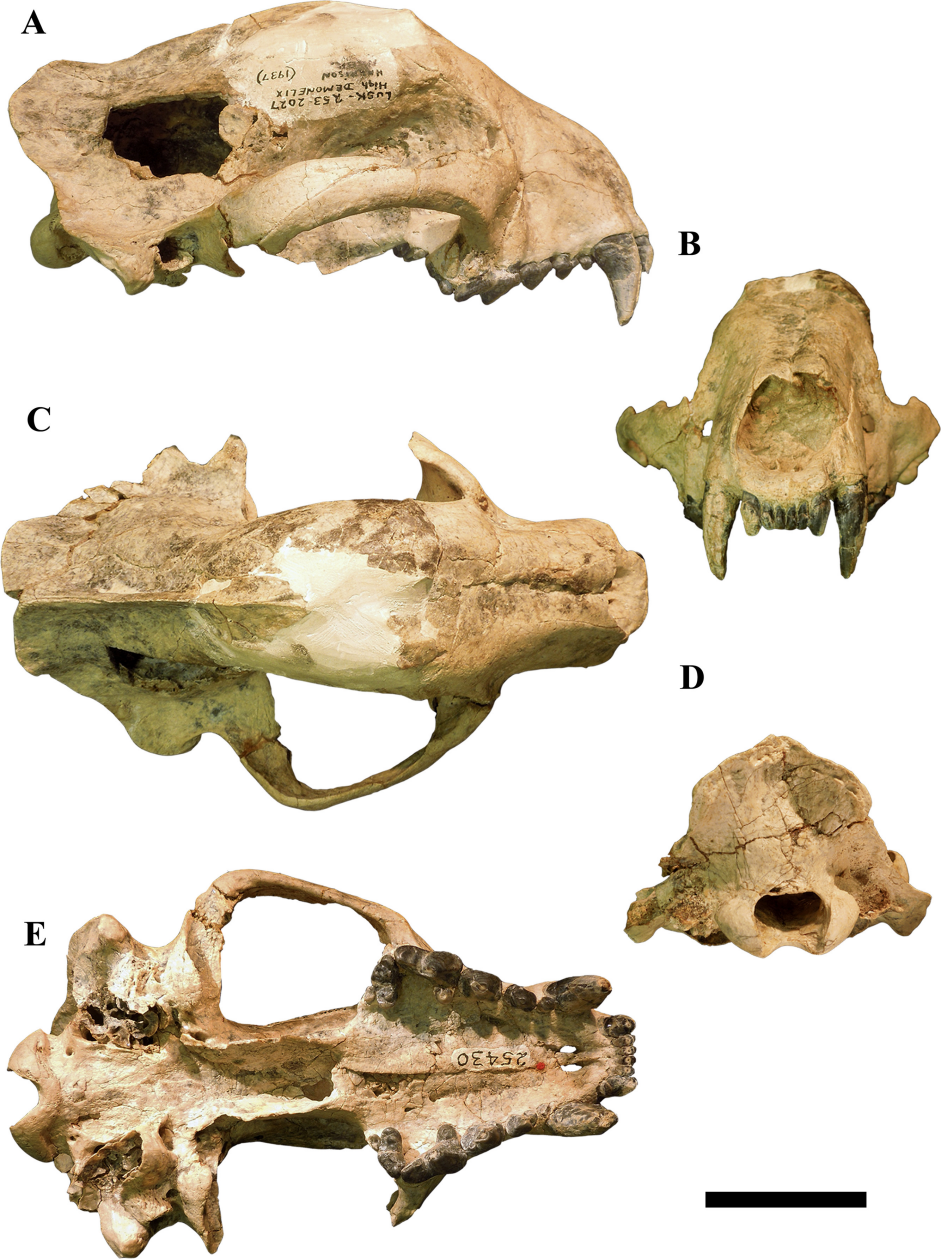
Cranium F:AM 25430 of *Megalictis ferox*. A lateral view; B rostral view; C dorsal view; D caudal view; E ventral view. Scale bar equals 5 cm.

**Fig 2:**
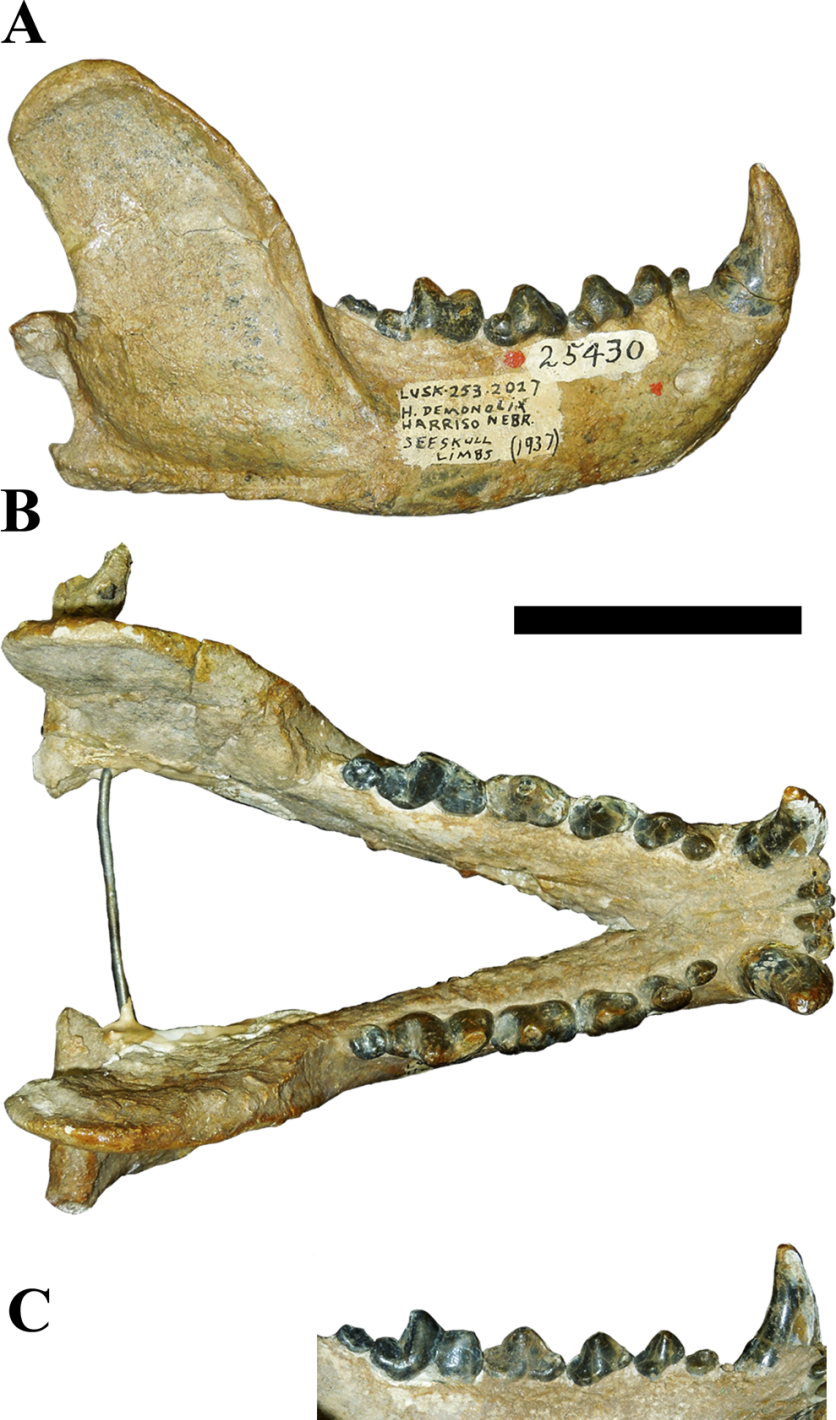
Mandible F:AM 25430 of *Megalictis ferox*. A Right mandible lateral view; B occlusal view; C Left mandible lingual view of lower dentition. Scale bar equals 5 cm.

**Fig 3:**
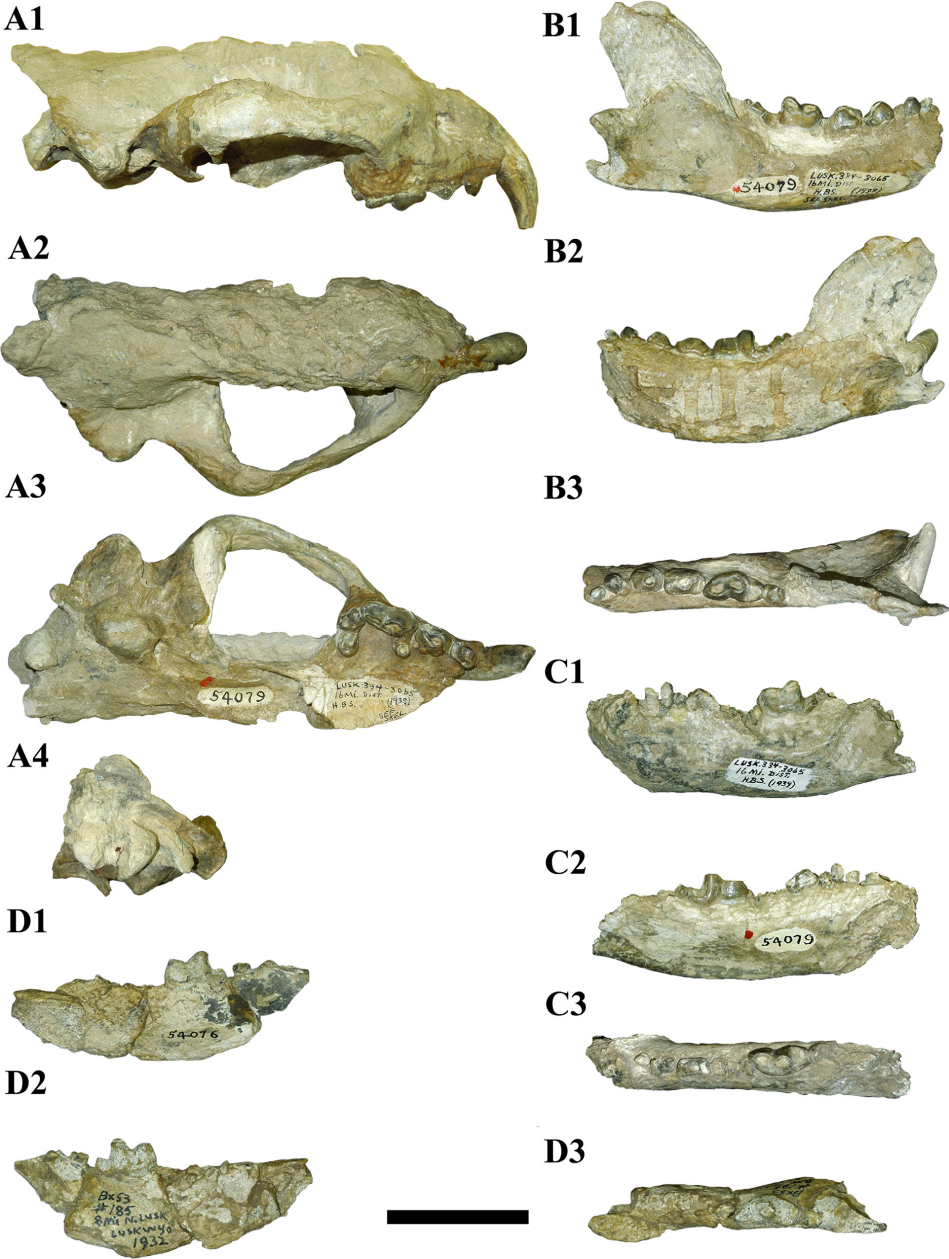
Cranium and mandibles remains of F:AM 54079 and AMNH 54076 of *Megalictis ferox*. A1–4 Cranium F:AM 54079, lateral view (A1), dorsal view (A2), ventral view (A3), and caudal view (A4); B1–3 right hemimandible F:AM 54079, lateral view (B1), medial view (B2), and occlusal view (B3); C1–3 left hemimandible F:AM 54079 lateral view (C1), medial view (C2), and oclussal view (C3); D1–3 right hemimandible of AMNH 54076, lateral view (D1), medial view (D2), and occlusal view (D3). Scale bar equals 5 cm.

**Fig 4:**
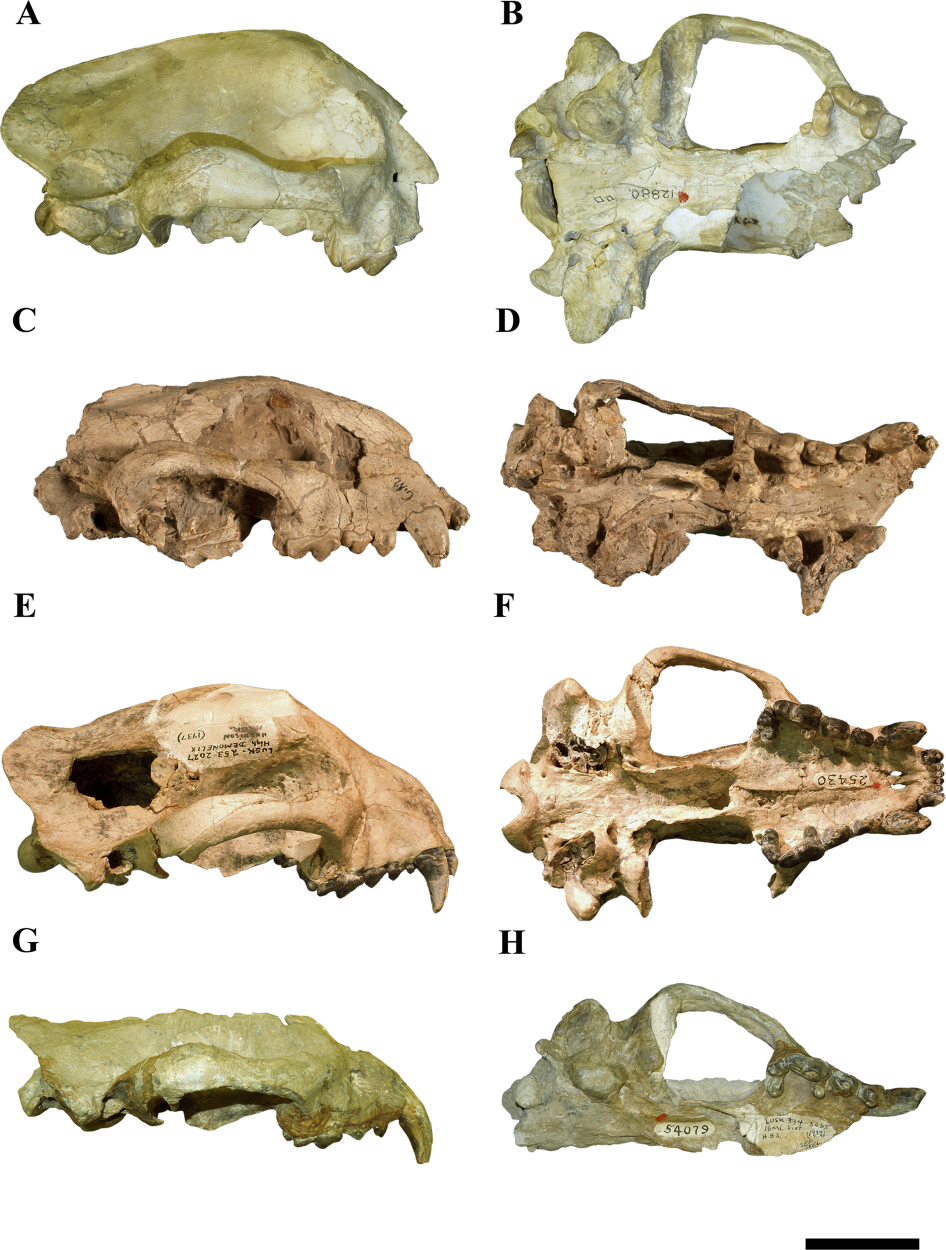
Crania of *Megalictis ferox* illustrating size differences. A, and B *Megalictis ferox* holotype AMNH 12880, lateral view (A), ventral view (B); C, and D *Megalictis ferox* CM 1590 (genotype of *Aelurocyon brevifacies*), lateral view (C), ventral view (D); *Megalictis ferox* F:AM 25430 lateral view (E), ventral view (F); G, and H *Megalictis ferox* F:AM 54079 lateral view (G), ventral view (H). Scale bar equals 5 cm. C and D courtesy of the Carnegie Museum of Natural History.

Dental nomenclature follows Ginsburg [17] and Smith and Dodson [18]. Anatomical descriptions are based primarily on Scapino [19], Turnbull [20], Barone [21, 22], Waibl et al. [23], Evans and de Lahunta [24, 25], and Hartstone-Rose et al. [26]. The terminology conforms to the standard of the *Nomina anatomica Veterinaria* [23] with the exception of the *masseter* and *temporalis* muscle complexes for which we follow Hartstone-Rose et al. [26]. The *Megalictis* material (Figs 1–4) has been compared to all the other material of *Megalictis* and *Paroligobunis* on the basis of published descriptions, figures, measurements and photographs. We have re-measured the dentition of AMNH 12880 and 22632 (cast of CM 1590) measured initially by Matthew [1] and Peterson [5] and completed the measures of *Paroligobunis petersoni* Loomis, 1932 [27] using a cast TMM 40966–1. Measurements were made using Mitutoyo Absolute digital calipers to the nearest 0.1 mm (Tables 1 and 2).

**Table 1:** Upper tooth measurements (in mm) of *Megalictis ferox*.

	C		P1		P2		P3		P4		M1		M2	
Taxa	L	W	L	W	L	W	L	W	L	W	L	W	L	W
*M*. *ferox* AMNH-22632*	17	13.4	-	-	10.6	6.3	15.8	9.9	23.4	16.1	7.7	18.4	-	-
*M*. *ferox* AMNH 12880	-	-	-	-	-	-	-	-	24.5	18.5	8.5	19.6	4.4	6.3
*M*. *ferox* F:AM 54079	14.6	10.9	-	-	11.1	8.4	14.1	9.9	21.6	17.3	7.4	18.7	-	-
*M*. *ferox* F:AM 25430^a^	14.3	14.3	5.4	4.9	10.6	6.9	14.2	9.9	21.8	15.7	8.2	17.1	3.1	5.5
*M*. *ferox* F:AM 25430^b^	14.0	14.0	5.0	4.6	10.6	7.6	14.3	9.7	21.8	16.0	8.3	17.7	3.0	5.3

*Cast of CM 1590

^a^Left dentition

^b^Right dentition

L = Length

W = width

**Table 2:** Lower tooth measurements (in mm) of *Megalictis ferox*, *Megalictis simplicidens*, *Megalictiss frazieri*, and *“Megalictis” petersoni*.

	c		p1		p2		p3		p4		m1		m2	
Taxa	L	W	L	W	L	W	L	W	L	W	L	W	L	W
*M*. *ferox* AMNH-22632**	-	-	-	-	10.2	7.1	14.0	9.4	16.6	9.5	21.3	10.1	7.0	5.7
*M*. *ferox* AMNH 12880	-	-	-	-	-	-	-	-	-	-	24.0*	10.9*	7.3	6.1
*M*. *ferox* F:AM 54079^a^	-	-	4.9	4.4	-	-	-	-	-	-	20.6	9.9	-	-
*M*. *ferox* F:AM 54079^b^	-	-	5.0	4.2	9.7	7.0	10.9	8.7	14.4	9.1	20.7	10.0	8.3	6.4
*M*. *ferox* F:AM 25430^a^	14.3	11.7	5.8	4.4	9.4	6.5	12.4	8.4	15.7	8.8	19.1	9.6	6.4	6.1
*M*. *ferox* F:AM 25430^b^	-	-	6.1	4.3	9.2	6.5	12.2	8.7	15.4	8.7	18.9	9.6	7.0	6.0
*M*. *ferox* AMNH 54076	-	-	-	-	-	-	-	-	-	-	21.3	9.9	7.0	5.3
*M*. *simplicidens* CM 1553^c^	11.2	8.2	-	-	8.8	5.7	9.7	6.9	11.6	7	16.4	7.6	-	-
*M*. *frazieri* UF 23928 ^c^	9.5	7.5	-	-	7.4	4.8	8.1	5.6	10.8	6.1	15.7	7.1	-	-
*“M”*. *petersoni* TMM 40966–1 ***	-	-	2.3	2.1	4.9	3.4	5.7	3.7	6.8	3.9	10.2	5.2	3.6	3.4

*Approximated

**Cast of CM 1590

*** Cast of ACM 2011

^a^Left dentition

^b^Right dentition

^c^Frailey, 1978 [28]

L = Length

W = width

### Studied Material

F:AM 25430 (Figs 1 and 2, S1 Video): relatively complete skull with I1-3, C, P1-4 and M1-2, missing only its left zygomatic arch, a broken frontal area plus a portion of the right parietal region missing and filled with plaster, a hole in its right parietal bone, and a complete mandible with i1-3, c, p1-4 and m1-2; F:AM 54079 (Fig 3, S2 Video): right side of a partial skull without the premaxilla, with worn C, P2-M1 and partial mandibles with a nearly complete right one with c-m2 and a broken mandibular symphysis and a left one just with the mandibular corpus preserved and a broken p2, and a complete both p3 and m1; AMNH 54076 (Fig 3): partial mandibular corpus with m1-2.

The extant specimens used for comparison and contextualization in this paper were the mustelids *Gulo gulo* (n = 20), *Taxidea taxus* (n = 8), *Mellivora capensis* (n = 21), *Pekania pennanti* (n = 5), *Eira barbara* (n = 8), *Martes martes* (n = 4), and *Mustela putorius* (n = 5), the procyonids *Bassariscus astutus* (n = 1), *Procyon lotor* (N = 5) and *Nasua nasua* (N = 5), the mephitid *Mephitis mephitis* (n = 1) and the canid *Canis lupus* (n = 5) (S1 Table). To provide a comparison to giant mustelids we used the holotype of *Megalictis ferox* Matthew, 1907 [1] (AMNH-12880) (Fig 4A and 4B, S3 Video), and a cast of the holotype of *Aelurocyon brevifacies* Peterson, 1907 [5] AMNH-22632 (CM 1590) (S4 Video) both housed at AMNH; the holotype of *Paroligobunis petersoni* Loomis, 1932 [27] (ACM 2011) from Wyoming, USA housed at ACM and a cast of *P*. *petersoni* TMM 40966–1 housed at TMM; the holotype of *Paroligobunis frazieri* Frailey, 1978 [28] (UF 23928) from Florida, USA (S5 Video) housed at UF; *Paroligobunis simplicidens* (Peterson, 1907, 1910) [5, 29] (CM 1553 and CM 2389) from Nebraska, USA housed at CM, and a cast of the holotype of *Paroligobunis simplicidens* CM 1553 housed at UF (S5 Video); the holotype of *Oligobunis crassivultus* Cope, 1879 [30] (AMNH 6903) from Oregon, USA housed at AMNH; *Eomellivora piveteaui* Ozansoy, 1965 [31] from Cerro de los Batallones, Spain [32] housed at MNCN; the holotype of *Eomellivora ursogulo* (Orlov, 1948) [33] from Grebeniki, Ukraine housed at PIN; *Plesiogulo monspesulanus* Viret, 1939 [34] from Langebaanweg, South Africa [35] housed at SAM-PQL; *Plesiogulo crassa* Teilhard, 1945 [36] from Perivolaki, Greece [37] housed at LGPUT; and a cast of the holotype of *Ekorus ekakeran* Werdelin, 2003 [13] from Lothagam, Kenya from the personal research collection of L. Werdelin housed at NRM.

### Cladistic analysis

**Fig 5:**
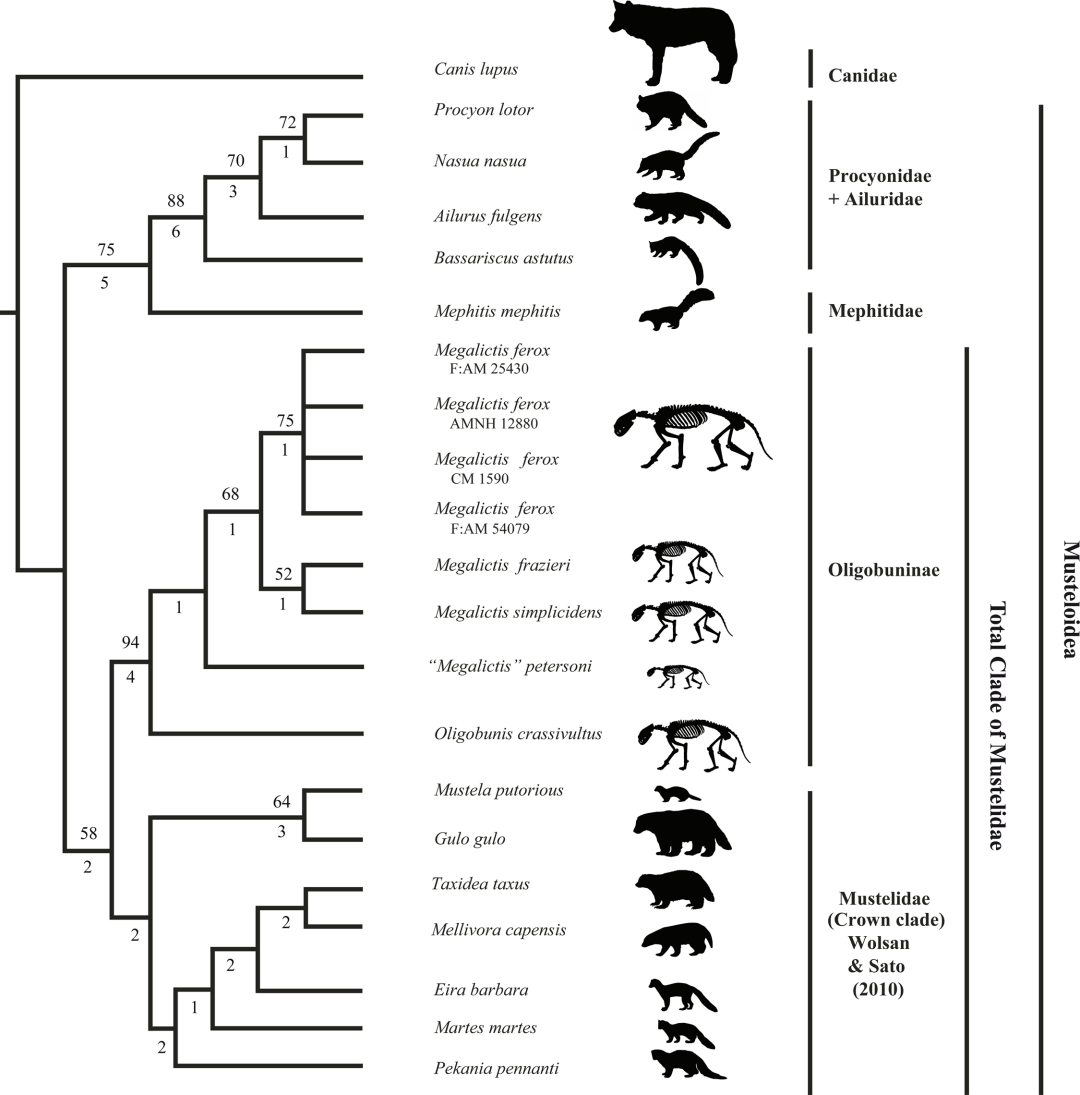
Phylogenetic relationships of *Megalictis* within Mustelidae. Searches were performed using the Branch and Bound and a Bootstrap analysis through 1000 replicates to test the clade support in the analysis. The outgroup was *C*. *lupus*. Strict consensus tree of 6 trees (Length 194 steps, consistency index (CI) = 0.41, retention index (RI) = 0.65) for knowing the relationships between the different specimens of *Megalictis ferox*, *Megalictis simplicidens*, *Megalictis frazieri*, “*Megalictis*” *petersoni*, *Oligobunis crassivultus*, and a sample of extant musteloids and a canid. Numbers below nodes are Bremer indices, and numbers above nodes are Bootstrap support percentages (only shown when ≥ 50). Character/taxa matrix is detailed in the S1–S3 Appendices. Silhouette of *Megalictis ferox* based on Hunt and Skolnick [7], silhouette of *Megalictis simplicidens*, *Megalictis frazieri*, “*Megalictis*” *petersoni* and *Oligobunis crassivultus* based on *Megalictis ferox* but rescaled according the size of the dentition.

In order to better understand the phylogenetic relationships of the oligobunines *Megalictis ferox* (AMNH 12880, CM 1590, F:AM 25430 and F:AM 54079), *M*. *simplicidens* (= *Paroligobunis simplicidens*) (CM 1553 and CM 2389), *M*. *frazieri* (*= Paroligobunis frazieri*) (UF 23928), “*M*.*” petersoni* (= *Paroligobunis petersoni*) (ACM 2011), and *Oligobunis crassivultus* (AMNH 6903), we have performed a cladistic analysis (Fig 5) including 18 taxa (*M*. *ferox* is represented in the analysis as 4 separate operational taxonomic units (OTU)) and 73 equally weighted and unordered craniomandibular characters (S1–S3 Appendices). Cladistic analysis was performed using in PAUP*4.0b10 [38]. The analysis was rooted using *C*. *lupus* as the outgroup.

### 3D models

Virtual models of the mandibles and skulls of *Megalictis ferox* (F:AM 25430, F:AM 54079, AMNH 12880 and AMNH 22632) as well as *Megalictis frazieri* UF 23928 and *Megalictis simplicidens* (cast of CM 1553) were derived by means of a 3D NextEngine HD laser surface scanner (S1–S6 Videos).

Virtual models of the mandibles and skulls of *Megalictis ferox* (F:AM 25430, F:AM 54079, AMNH 12880 and AMNH 22632) as well as *Megalictis frazieri* UF 23928 and *Megalictis simplicidens* (cast of CM 1553) were derived by means of a 3D NextEngine HD laser surface scanner (S1–S6 Videos).

## Systematic paleontology

Order Carnivora Bowdich, 1821 [39]

Suborder Caniformia Kretzoi, 1943 [40]

Family Mustelidae Fisher, 1817 [41]

Subfamily Oligobuninae Baskin, 1998 [2]

Genus *Megalictis* Matthew, 1907 [1]

Diagnosis: Large to giant size mustelid; robust mandible with a high, wide and distally curved ascending ramus; deep masseteric fossa with a stout crest that extends from the dorsal border of the coronoid process to below the m2; robust dentition; p1 present; p2–4 with distal cingula high-crowned; p4 relatively enlarged with mesial and distal accessory cuspids; m1 trigonid widened, with a strong lingual concavity between the paraconid and protoconid; m1 metaconid reduced to absent, present in the older and smaller forms and absent in the giant forms, m1 talonid low and narrow with a short, trenchant and labially located hypoconid; and m2 with reduced metaconid.

Type species: *Megalictis ferox* Matthew, 1907 p1.II, fig.1 [1]

Included species: *Megalictis simplicidens* (= *Paroligobunis simplicidens*) (Peterson, 1907) [5] and *Megalictis frazieri* (*= Paroligobunis frazieri*) (Frailey, 1978) [28].

Synonyms: Senior subjective synonym [7] of *Aelurocyon brevifacies* Peterson, 1907, p. 68 [5], “Upper Harrison Formation”, Sioux County, Nebraska and *Paroligobunis* Peterson, 1910 [29]. Hunt and Skolnick [7] synonymized *Megalictis*, *Aelurocyon*, and *Paroligobunis simplicidens* into a single, sexually-dimorphic chronospecies *M*. *ferox*. This hypothesis has been generally accepted (e.g., [3, 13, 42]).

### *Megalictis ferox* Matthew, 1907 [1]

*Aelurocyon brevifacies*, Peterson, 1907, p. 68. [5]

*Megalictis ferox*, Hunt and Skolnick, 1996 (pars). [7]

*Aelurocyon ferox*, Baskin, 1998, p. 156. [2]

Holotype: AMNH 12880, a partial reconstructed skull (Fig 4, S3 Video) with right P4, M1-2, a fragmented right mandible with almost complete coronoid process, m1 trigonid and m2, and very few postcranial remains figured by Matthew, 1907, p. 196, fig. 10–13, 15 [1].

Type Locality: Rosebud 22, Porcupine Butte, Black Bear Formation, Stanley County, South Dakota.

Other Localities: Rosebud 5, Porcupine Butte, Stanley County, South Dakota, USA (AMNH 12881); Niobrara Canyon Local Fauna, Sioux County, Nebraska, USA (CM 1590), “High Daemonelix beds”, Niobrara Canyon Local Fauna, Sioux County, Nebraska, USA (F:AM 25430); J-M District, South of Lusk, Goshen County, Wyoming, USA [6]; “high brown sand”, 16 Mile District, Goshen County, Wyoming, USA (F:AM 54079); 8 North of Lusk, Goshen County, Wyoming, USA (F:AM 54076).

Age: Upper part of the Anderson Ranch Formation and its equivalents, South Dakota, Nebraska, and Wyoming, late late Arikareean (Ar4), 22.7–18.5 mya [4] Early Miocene.

Diagnosis: Baskin [2] diagnosed of *Aelurocyon brevifacies* (which he considered the senior subjective synonym of *Megalictis ferox* because of the presumed earlier publication date at the time he submitted the chapter). New or revised characters follow. *Megalictis ferox* is the largest of the oligobunines; coronoid process high and caudally curved; enlarged masseteric fossa with a robust crest extending from the dorsal border of the coronoid process to below the m2; laterocaudal area of the ventral edge of the mandibular corpus laterally projected; P2 with distal accessory cusp; robust P3; robust P4 with strong parastyle and protocone; P4 carnassial notch present; M1 with enlarged stylar area; M2 with paracone and protocone; p2–4 with high-crowned distal cingula; p3 with mesial and distal accessory cuspid; p4 relatively enlarged with presence of mesial accessory cuspid and stout distal accessory cuspid; m1 trigonid widened; m1 with strong lingual concavity between paraconid and protoconid; m1 protoconid higher than paraconid; m1 hypoconid short, trenchant and buccally located; m1 with a lingual cingulum in the entoconid position; m2 reduced with metaconid.

Differential Diagnosis: *Megalictis ferox* differs from *M*. *simplicidens*, *M*. *frazieri*, *“M*.*” petersoni* and *Oligobunis crassivultus* in its larger size, m1 without metaconid and m1 talonid with a closed lingual morphology with a lingual cingulum between the metacristid and entocristid. Additionally, it differs from *M*. *simplicidens* and *M*. *frazieri* in having a higher and more robust mandibular symphysis, a reduced p2 and a more robust p4 and m1. It further differs from *“M*.*” petersoni* in much larger size and p3–4 with mesial accessory cuspids. It further differs from *Oligobunis crassivultus* in having a more rectangular P2, smaller M1 than P4, enlarged M1 stylar area, higher paracone than metacone on the M1, reduced p2, p2–3 high-crowned distal cingula, more developed p3 distal accessory cuspid, relatively enlarged p4, and higher protoconid than paraconid on the m1.

Comments: Specimens that can be referred to *M*. *ferox s*. *s*. are from the latest Arikareean (Ar4) upper part of the Anderson Ranch Formation and its equivalents.

#### F:AM 25430

A nearly complete skull with I1-3, C, P1-4 and M1-2 (Fig 1, S1 Video) and a complete mandible with i1-3, c, p1-4 and m1-2 (Fig 2, S1 Video). The left zygomatic arch is missing. Part of the frontal and a region of the right parietal bones are missing and filled with plaster. There is a subrectangular and anthropogenic hole in its right parietal bone located above the most caudal area of the zygomatic arch.

Locality: “High Daemonelix beds”, Anderson Ranch Formation, Niobrara Canyon Local Fauna, Sioux County, Nebraska, USA.

Age: Late Arikareean (Ar4).

Skull and upper dentition: The very well preserved skull F:AM 25430 (Fig 1) has a basicranial length of 189.5 mm. It is slightly domed dorsally at the frontal bone, the bullae are broken and the left zygomatic arch is missing. In general terms the skull is high, domed with a short rostrum and high snout (Fig 1A). The nasal aperture is large (Fig 1B), and the crushed nasal bones are robust. They are crushed in the mid-sagittal plane and anteriorly the left nasal bone is partially above the right one. The reconstructed frontal region is quite domed. The interorbital region is broad. The postorbital processes are absent. The moderately developed infraorbital foramen is rounded and located above the distal accessory cusp of the P3. The rostral margin of the orbit ends at the level of the distal margin of the P4 paracone. The orbits are large and rounded. The lacrimal foramen is rounded and relatively large. The sagittal crest is moderately developed and extends caudally where it divides into the nuchal crests, forming a Y-pattern (Fig 1A, 1C and 1D). In lateral view, the outline of the skull is convex in the temporal region and concave between the temporal bone and nuchal crests.

The zygomatic arches are robust, especially caudally near the glenoid cavity. Both M. *masseter pars superficialis* and M. *masseter pars profunda* have their origin on the ventrolateral side of the zygomatic arch. The frontal processes of the zygomatic arches are triangular and dorsoventrally high.

Ventrally (Fig 1E), the incisive foramina are preserved. The palate is broad and expanded mediolaterally between the P4–M2. The posterior border of the palatine is expanded caudally behind the molars. The pterygoid region and the hamulus pterygoideus processes are relatively well preserved. The hamulus pterygoideus processes are large and caudally expanded (Fig 1E). The foramen ovale is located in line with the glenoid fossa. The alisphenoid canal is absent. The glenoid fossa is relatively strong. The auditory bullae are large and swollen. The external auditory meati are rounded (Fig 1A). The ventral wall of the auditory bullae has been partially destroyed, and the tympanic chamber is exposed. The postglenoid foramen is large, rounded and located caudally to the postglenoid process and medially to the external auditory meatus. The rostral foramen lacerum or external carotid foramen is a large double foramen located on the rostromedial corner of the auditory bullae. The caudal carotid foramen is almost hidden and is located in line with the external auditory meatus, midway along the medial margin of the auditory bullae. The large rounded caudal foramen lacerum is located on the caudal-most corner of the skull. The suprameatal fossa is absent. The condyloid foramen is located caudally to the caudal foramen lacerum and is clearly separated from it. The stylomastoid foramen is not preserved. The occipital condyles are strong and their dorsal parts are broader than the ventral ones. The foramen magnum is large and subquandrangular (Fig 1D). The mastoid process is highly expanded (Fig 1C and 1E); measuring 106.1 mm in width. The caudal area of the skull is very broad. The nuchal crest has a great caudal development. Its dorsal part is projected caudally. In dorsal view the ventral parts of the nuchal crest in conjunction with the mastoid process are laterally widened, which creates large attachment areas for M. *zygomatic temporalis* on the dorsal side (Fig 1C) and M. *obliquus capiti cranialis* on the caudal side (Fig 1D). The mastoid process is robust and is situated caudal to the external auditory meatus. The supraoccipital bone is very enlarged. The paroccipital process is not preserved.

The upper dentition (3/1/4/2) is preserved in its entirety (Fig 1E). The tooth rows are rectilinear between C–P4. The upper incisors are set in a straight line and show a large occlusal wear facet to the same extent as the wear on the premolars. I3 is much larger than I1 and I2 (Fig 1A and 1B). It is a caniniform tooth with a single cusp, and has a distal wear facet due the contact with the c. The crown displays a lingual curve, and a lingual cingulum. A diastema of 18 mm separates I3–C (Fig 1E). The C is robust, and oval in cross-section. P1–4 have strong cingula. The P1 is reduced, single-cusped and rounded. The P2 (Fig 1A and 1E) is narrow in the middle of the tooth. The distal part is widened. It has a low mesial and two distal accessory cusps. The main cusp is high and mesially oriented. The P3 is subrectangular in occlusal view. It is a massive tooth with a small mesial accessory cusp and a more developed distal one (Fig 1A and 1E). It widens in the buccodistal area. The P4 has a low parastyle located on the mesial cingulum. The paracone is the highest and largest cusp, occupying over half of the total length of the tooth; there is a carnassial notch between it and the parastyle. The protocone is subconical, robust, and projected distolingually, but in line with the parastyle. There is a concavity in the buccal wall between the paracone and the metastyle. The latter is low, with a swollen distal region. The M1 is enlarged buccolingually and reduced mesiodistally. There is an expansion at the level of the paracone and metacone, and a mesiodistal constriction in the middle zone of teeth (Fig 1E). The paracone is more developed than the metacone. It has an enlarged parastylar shelf. The protocone is located in the middle of the lingual corner. It is large, stout and crest-like. There is a small crest-shaped paraconule in contact with the protocone. It has a lingual platform rounded the protocone with a small hypocone in the messiolingual corner. The M2 is very reduced and oval (Fig 1E). It has a paracone and no metacone. The protocone is as developed as the paracone.

Mandible and lower dentition: The mandible of F:AM 25430 is very robust (Fig 2A and 2B). It has a total length of 149.0 mm. The tooth row is slightly convex and is aligned with the articular process. The mandibular corpus is high and robust. The ventral margin is convex at the level of the m1. There is single rounded mental foramen under p2. The ascending ramus is tall and rostrocaudally broad (Fig 2A). Its tip is distally oriented. The coronoid process is laterally rotated with an angle of ~75 degrees, compared to the articular process. There is a robust crest from the dorsal border of the coronoid to beneath the m2 where the tendon of the M. *temporalis* is attached. This area is especially enlarged and laterally projected around the area of the m2 (Fig 2A and 2B). The masseteric fossa is large and deep. Its rostral margin lies at the level of talonid of m1, and ventrolaterally is limited by a strong area where the M. *masseter pars superficialis* and M. *masseter pars profunda* insert. The articular process is large and robust. The angular process is robust and shows a medial crest for the muscular attachment of the M. *pterygoideus medialis*.

The lower dentition (3/1/4/2) is also preserved in its entirety (Fig 2). The lower incisors are heavily worn. The canine is large, stout and markedly curved distally (Fig 2A and 2B). It has a swollen base and is oval in cross-section. The p1 is oval, single-cusped and distally wide (Fig 2B). The p2–4 are stout, subrectangular and wider distally. These premolars have strong cingula at their bases, and the distal cingula are high-crowned. The p2 has a single messially-located cuspid. The p3 has a low mesial accessory cuspid and a more developed distal one. The p4 is the largest lower premolar and has more strongly developed mesial and distal accessory cuspids. The m1 is a relatively short and stout tooth (Fig 2). The very robust trigonid occupies almost three fourths of the total length of the tooth, with the greatest width at the base of the protoconid. The paraconid is lower than the protoconid and there is no metaconid. The m1 shows a markedly lingual concavity in the base of the crown between the trigonid cuspids (Fig 2B). The stout talonid lacks an entoconid. The hypoconid is low, trenchant and buccally located. There is a smooth cristid from the top of the protoconid to the hypoconid that encloses a deep lingual depression (Fig 2C). The m2 is rounded and low (Fig 2B). The paraconid is low, and located in the mesial corner. The protoconid is the highest cuspid, located buccally in the middle of the tooth. The metaconid is situated over the lingual corner. It is less developed than the protoconid. The hypoconid is low and located in the distal corner. There is a cingulum around the whole tooth.

#### F:AM 54079

Partial skull with worn C, P2–M1 and partial mandible with worn p1–4 and m1–2 (Fig 3A1–4, 3B1–3 and 3C1–3, S2 Video).

Locality: “High brown sand”, 16 Mile District, Anderson Ranch Formation, Goshen County, Wyoming, USA.

Age: Late Arikareean (Ar4), Early Miocene.

Skull and upper dentition: The skull F:AM 54079 (Fig 3A1–4) only preserves its right side. It has a maximum length of 180.2 mm. The premaxilla is missing, so the basicranial length is unknown. In general terms, F:AM 54079 resembles F:AM 25430 (Fig 1). The frontal bone and dorsal area of the parietal bone are absent (Fig 3A2). The zygomatic arches are more robust than those of F:AM 25430, especially in the rostral and the central part of the arches, and the origin of M. *masseter pars superficialis* and M. *masseter pars profunda* are also more developed. However, the frontal processes of the zygomatic arches are lower than those of F:AM 25430. The glenoid fossa is stout with a very well developed postglenoid process (Fig 3A3). The complete right auditory bulla is large and swollen. The external auditory meati are rounded. The postglenoid foramen, the rostral foramen lacerum and the foramen ovale are similar to those of F:AM 25430. The mastoid process is also robust and expanded. The right occipital condyle is preserved but the caudodorsal area of the skull is not. The paroccipital process is triangular, stout and caudally oriented (Fig 3A1 and 3A3–4).

C, P2–4 and are preserved. The P1 is missing. They are more worn than are those of F:AM 25430. The C has a large lingual wear facet. The morphology of P2–4 (Fig 3A3) is almost identical to that of F:AM 25430. The P3 is more quadrangular than that of F:AM 25430, but the mesiolingual corner of the P3 is missing. The P4 paracone, protocone and metastyle are greatly-worn (Fig 3A3). The M1 (Fig 3A3) has the same development of the cusps as that found in F:AM 25430, and shows a very similar morphology as that of AMNH 12880. The M2 and its alveoli are not preserved.

Mandible and lower dentition: The right hemimandible (Fig 3B1–3) has a fragmented corpus that is missing its symphyseal end but includes a complete ascending ramus with p1–4 and m1–2. Its morphology is almost identical to that of F:AM 25430. The left hemimandible (Fig 3C1–3) is missing its ascending ramus but includes a complete mandibular corpus, a complete p1, a fragmented p2, a highly worn p3, a complete m1 and a fragmented m2. The p1–4 and m1 are almost identical to those of F:AM 25430 though there is more substantial occlusal wear in p2–4 and m1 than in F:AM 25430. The m2 is oval and has a more developed metaconid than the m2 of F:AM 25430.

#### AMNH 54076

Right partial hemimandible with m1–2 (Fig 3D1–3).

Locality: 8 North of Lusk, Goshen County, Anderson Ranch Formation, Wyoming, USA.

Age: Late Arikareean (Ar4), Early Miocene.

Mandible and lower dentition: AMNH 54076 is a fragmented mandibular corpus missing its symphysis (Fig 3D1–3). It has roots for p2–3, and complete m1–2. The mandibular corpus is high and robust. The m1 is identical to those of F:AM 54079 and F:AM 25430. It has a stout trigonid, and a low talonid composed of a trenchant hypoconid, lingually located and a lingual depression. The m2 is rounded and low. It has a distinguishable protoconid and metaconid, and a continuous basal cingulum.

## Discussion

Matthew [1] described and named the first specimens of *Megalictis ferox*. The holotype is a fragmentary and reconstructed skull (Fig 4A and 4B, S3 Video), a partial mandible and some postcranial remains of a single individual (AMNH 12880). He [1] also described a second specimen (AMNH 12881) based on postcranial remains. Both individuals were found in two nearby localities (Rosebud 22 and Rosebud 5 respectively) at Porcupine Butte, South Dakota, USA, from the late late Arikareean (Ar4) Black Bear Formation. One week later [7], Peterson [5] named *Aelurocyon brevifacies* (CM 1590) for the remains of a giant mustelid from the upper part of the Anderson Ranch Formation in Niobrara Canyon, Sioux County, Nebraska, based on more complete craniomandibular fossils (Fig 4C and 4D, S4 Video). Riggs [6] studied a large sample of Ar4 postcranial and some cranial material he termed *A*. *brevifacies* from the JM-District, south of Lusk, Wyoming, and some postcranial fossil of *M*. *ferox* from the Anderson Ranch Formation. Based on these and specimens from Beardog Hill, Agate Fossil Beds National Monument, Sioux County, Nebraska that had been assigned to *Paroligobunis simplicidens* [5, 29], Hunt and Skolnick [7] synonymized the oligobunines *Megalictis ferox*, *Aelurocyon brevifacies*, and *Paroligobunis simplicidens* into a single chronospecies *M*. *ferox*. They [7] interpreted the differences observed in these three named taxa as attributable to individual and sexual variation and a slight degree of evolution over time. This hypothesis has been accepted by several authors (e.g., [3, 13, 42]).

Hunt and Skolnick [7] did not consider the other two species referred to *Paroligobunis*: the small *P*. *petersoni* Loomis, 1932 [27] and *P*. *frazieri* Frailey, 1978 [28]. As discussed below, we consider the material referred to both *P*. *simplicidens* and *P*. *frazieri* to be valid species: *Megalictis frazieri* and *M*. *simplicidens*.

The results of the cladistic analysis indicate that the specimens we assign to *M*. *ferox* form a monophyletic group (Fig 5). We agree with Hunt and Skolnick [7] in that *M*. *ferox* and *A*. *brevifacies* are the same taxon, and that *M*. *ferox* has priority. Morphologically, the specimens F:AM 54079, F:AM 25430 and AMNH 54076, as well as CM 1590 and AMNH 12880, are practically identical to each other (Figs 1–4). F:AM 54079 differs from F:AM 25430 and CM1590 in having a more robust p3 and a relatively longer m2. CM 1590 has a reduced lingual expansion of P3 and a stronger parastyle of P4 than F:AM 54079, F:AM 25430 and AMNH 12880. The morphology of F:AM 25430 is clearly different from the skull of AMNH 12880, and shows that the reconstructed parts of the latter were incorrect, in which the temporal, frontal and a part of the zygomatic arch bones are misinterpreted (Fig 4A and 4B). F:AM 25430 allows us to complete the knowledge about the morphology of the skull of *M*. *ferox* and showing that the holotype of *M*. *ferox* (AMNH 12880) and the holotype of *A*. *brevifacies* (CM 1590) belong to the same species. Consequently, F:AM 54079, F:AM 25430 and AMNH 54076 should be assigned to *M*. *ferox*. We agree with Hunt and Skolnick [7] that the difference observed in the specimens of *M*. *ferox* can be explained by intraspecific variability (sexual dimorphism and intrapopulational differences) or small temporal differences.

*Megalictis ferox* (Figs 1–4) is characterized by several traits: long external auditory meatus; high and caudally curved coronoid process; enlarged masseteric fossa with a robust crest from the dorsal border of the coronoid process to just beneath the m2; latero-caudal area of the ventral edge of the mandibular corpus is laterally projected, with the ventral edge of the angular process also laterally projected; I3 is enlarged; P2 with a distal accessory cusp; robust P3; robust P4 with carnassial notch; enlarged stylar area of M1, and a M2 with paracone and protocone differentiated; p2–4 distal cingula high-crowned; distal accessory cuspid on p3; relatively enlarged p4 with a stout mesial accessory cuspid; relatively stout m1 with a widened trigonid, a strong lingual concavity between the paraconid and protoconid, no metaconid, protoconid higher than paraconid, with a short, trenchant and buccally located hypoconid and a lingual rim in the entoconid position; reduced m2 with a metaconid.

**Fig 6:**
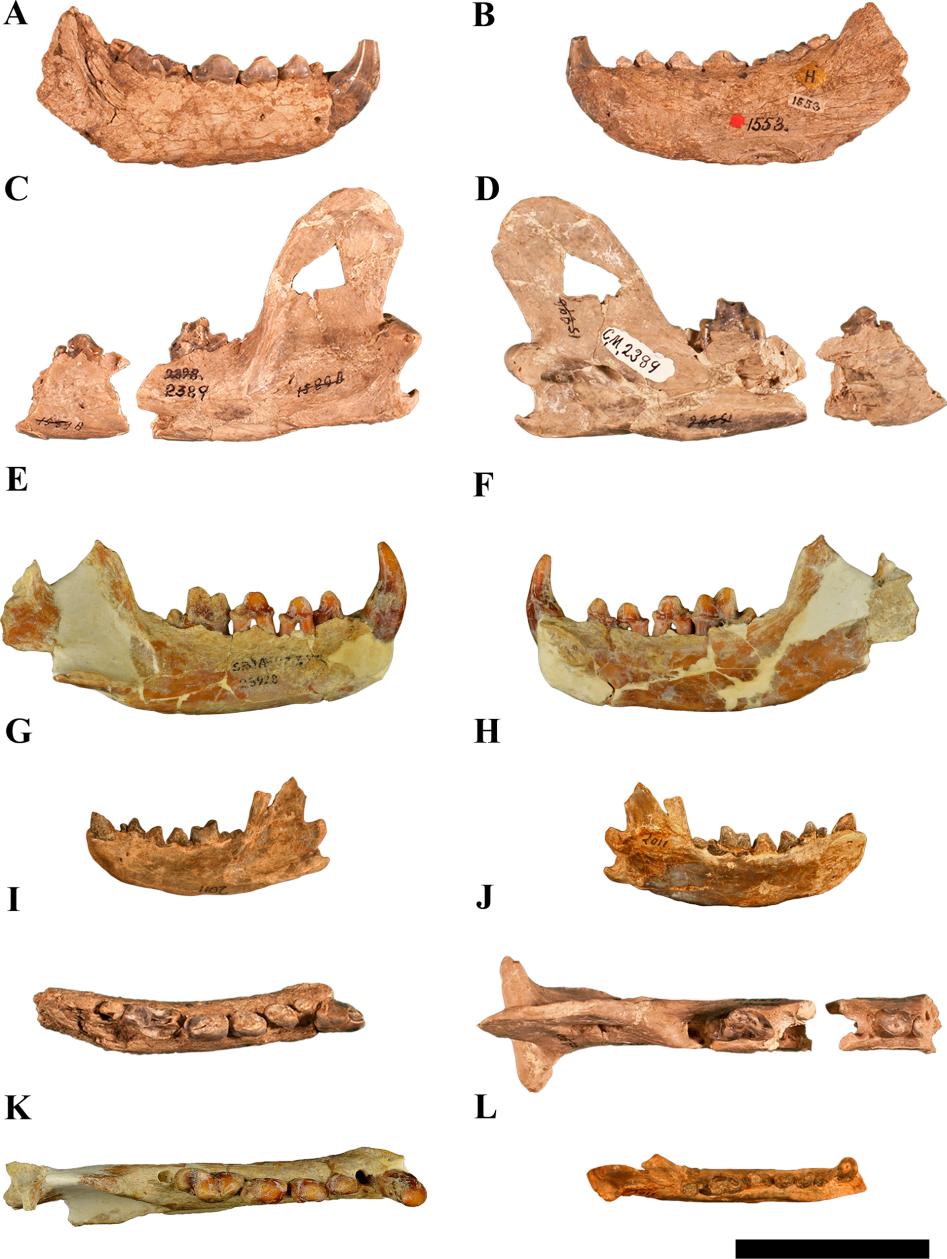
All remains of *Megalictis simplicidens*, *Megalictis frazieri* and “*Megalictis” petersoni* published. (A) *Megalictis simplicidens*, type specimen, CM1553 (Peterson, 1907) [5], lateral view of the mandible, (B) *Megalictis simplicidens* CM 1553 (Peterson, 1907) [5], medial view, (C) *Megalictis simplicidens* CM 2389 (Peterson, 1910) [29], lateral view of the mandible, (D) *Megalictis simplicidens* CM 2389 (Peterson, 1910) [29], medial view, (E) *Megalictis frazieri* (Frailey, 1978) [28], holotype UF 23928, lateral view of the mandible, (F) *Megalictis frazieri* (Frailey, 1978) [28], UF 23928, medial view, (G) “*Megalictis” petersoni* (Loomis, 1932) [27], holotype ACM 2011, lateral view of the mandible, (H) “*Megalictis” petersoni* (Loomis, 1932) [27] ACM 2011, medial view, (I) *Megalictis simplicidens* CM1553 (Peterson, 1907) [5], occlusal view, (J) *Megalictis simplicidens* CM 2389 (Peterson, 1910) [29], occlusal view, (K) *Megalictis frazieri* (Frailey, 1978) [28], UF 23928, occlusal view, (L) “*Megalictis” petersoni* (Loomis, 1932) [27] ACM 2011, occlusal view. Scale bar equals 5 cm. A-D, I and J courtesy of the Carnegie Museum of Natural History. E-F and K courtesy of the Florida Museum of Natural History. G-H and L, Beneski Museum of Natural History at Amherst College, courtesy of The Trustees of Amherst College.

All the three species that have been referred to *Paroligobunis* (Fig 6) are known from limited material. The genotype of *Paroligobunis*, *Megalictis simplicidens* (CM 1590, Peterson, 1907, 1910) [5, 29] comes from the “Agate Stock Farm”, Sioux County Nebraska. The exact locality is unknown and it is either from the Harrison Formation (Ar3) or the basal part of the Anderson Ranch Formation [7]. Additional material first referred to *P*. *simplicidens* [29] and later to *Megalictis ferox* [7] is from Quarry 3, Beardog Hill, Agate Fossil Beds National Monument, from the basal part of the Anderson Ranch Formation. The small *“M”*. *petersoni* (Loomis, 1932) [27] is from a locality near Van Tassel, Wyoming, “upper Harrison beds” (= Anderson Ranch Formation) and *P*. *frazieri* Frailey, 1978 [28] is from the SB-1A local fauna, Florida, latest Oligocene, early late Arikareean (Ar3). Hunt (in Tedford et. al, 2004:p. 205 [3]) recognized that “‘*Paroligobunis’ frazieri* is an earlier form preceding the late Arikareean species of *Megalictis*”.

There are no derived characters uniting the three named species of *Paroligobunis* that are not shared with *Megalictis* (S2 Appendix). Our phylogenetic analysis (Fig 5) shows that these three species are paraphyletic with *M*. *ferox*. The larger *P*. *frazieri* and *P*. *simplicidens* are both referred to *Megalictis*. The differences in morphology and size between the three species of *Megalictis* with respect to “*M*.*” petersoni* (Fig 6) suggest that “*M*.*” petersoni* could be excluded from the genus *Megalictis*.

*Megalictis simplicidens* and *M*. *frazieri* (Fig 6) resemble *M*. *ferox* in several characters, such as a high, wide and distally curved ascending ramus, and a deep masseteric fossa with a robust crest that extends from the dorsal border of the coronoid process to below the m2. Both taxa have a p1, the distal cingula of p2–4 are high-crowned, and the p4 is relatively enlarged with mesial and distal accessory cuspids. The m1 trigonid is widened, with a strong lingual concavity between the paraconid and protoconid, a low, and narrow talonid with a short, trenchant and labially located hypoconid, and a reduced m2 with presence of a metaconid. However they differ from *M*. *ferox* in having a non-reduced p2, the presence of a stout m1 metaconid, relatively more slender p4 and m1, m1 talonid with an open lingual morphology between the metacristid and entocristid, and a lower and more slender mandibular symphysis.

Hunt and Skolnick [7] partially described and measured some of the UNSM and CM specimens of *Megalictis* from the basal part of the Anderson Ranch Formation at Beardog Hill that we refer to *M*. *simplicidens*. Aside from their more primitive morphology (e.g., presence of a metaconid on m1), they are smaller than *M*. *ferox* from the upper Anderson Ranch Formation. The upper and lower dental measurements indicate a size similar to *G*. *gulo*.

*Megalictis frazieri* (Fig 6E, 6F and 6K) differs from *M*. *simplicidens* (Fig 6A–6D, 6I and 6J) in having a less massive mandible and a more distinctive distal cingulum with a higher crown in p2–4 than *M*. *simplicidens*. The c and p2 of *M*. *frazieri* are also more robust. The m1 hypoconid is higher and the talonid is relatively larger, slightly basined with a very low internal rim.

*“Megalictis” petersoni* (Fig 6G, 6H and 6L) differs from *M*. *simplicidens* and *M*. *frazieri* in the absence of mesial accessory cuspids on p3–4, a relatively stouter p4 with a shorter mesial part and a relatively more robust m1 with a taller and stouter metaconid.

**Fig 7:**
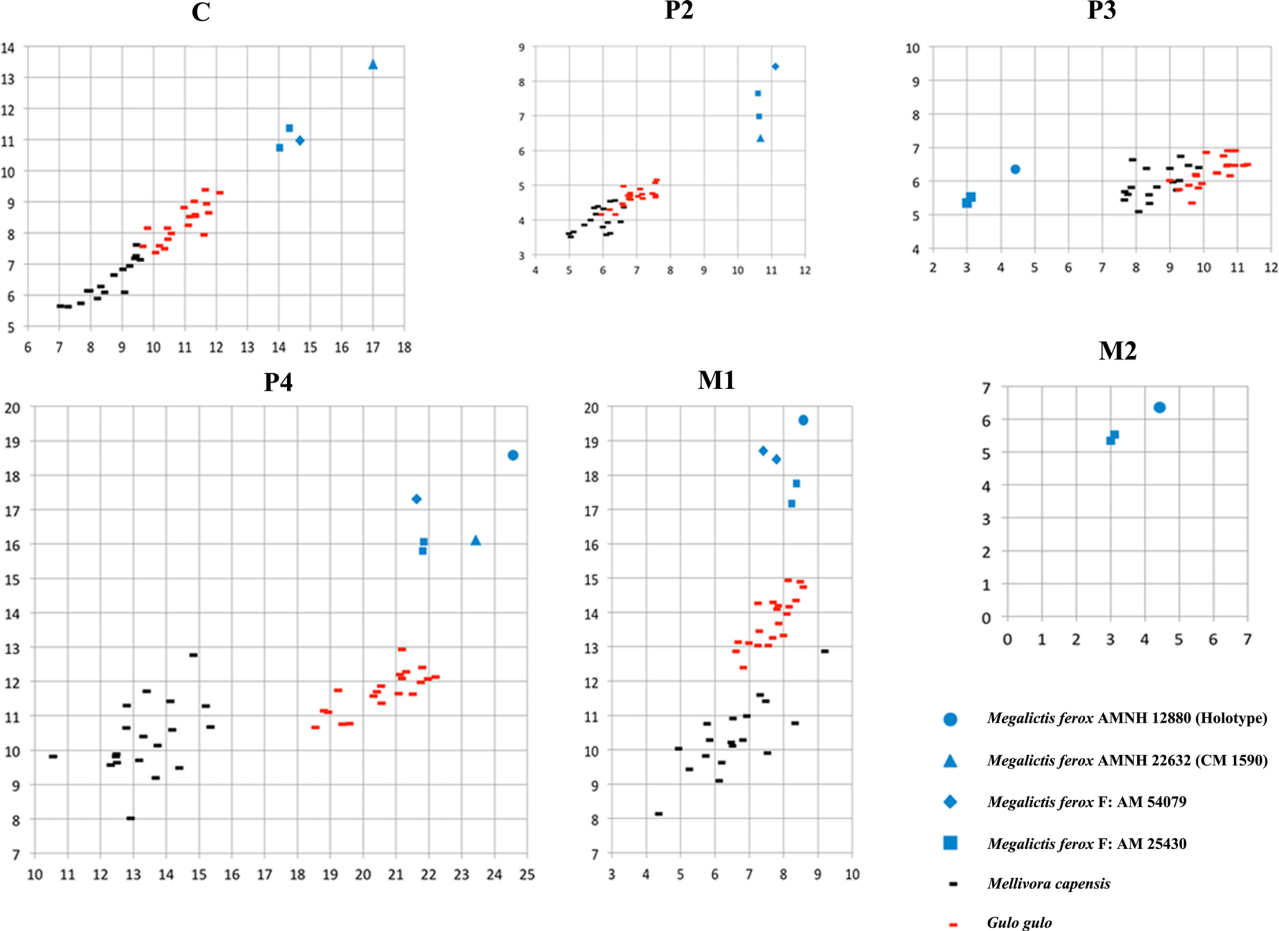
Relationships between lengths (L) and widths (W) of upper dentition in *Megalictis ferox*.

**Fig 8:**
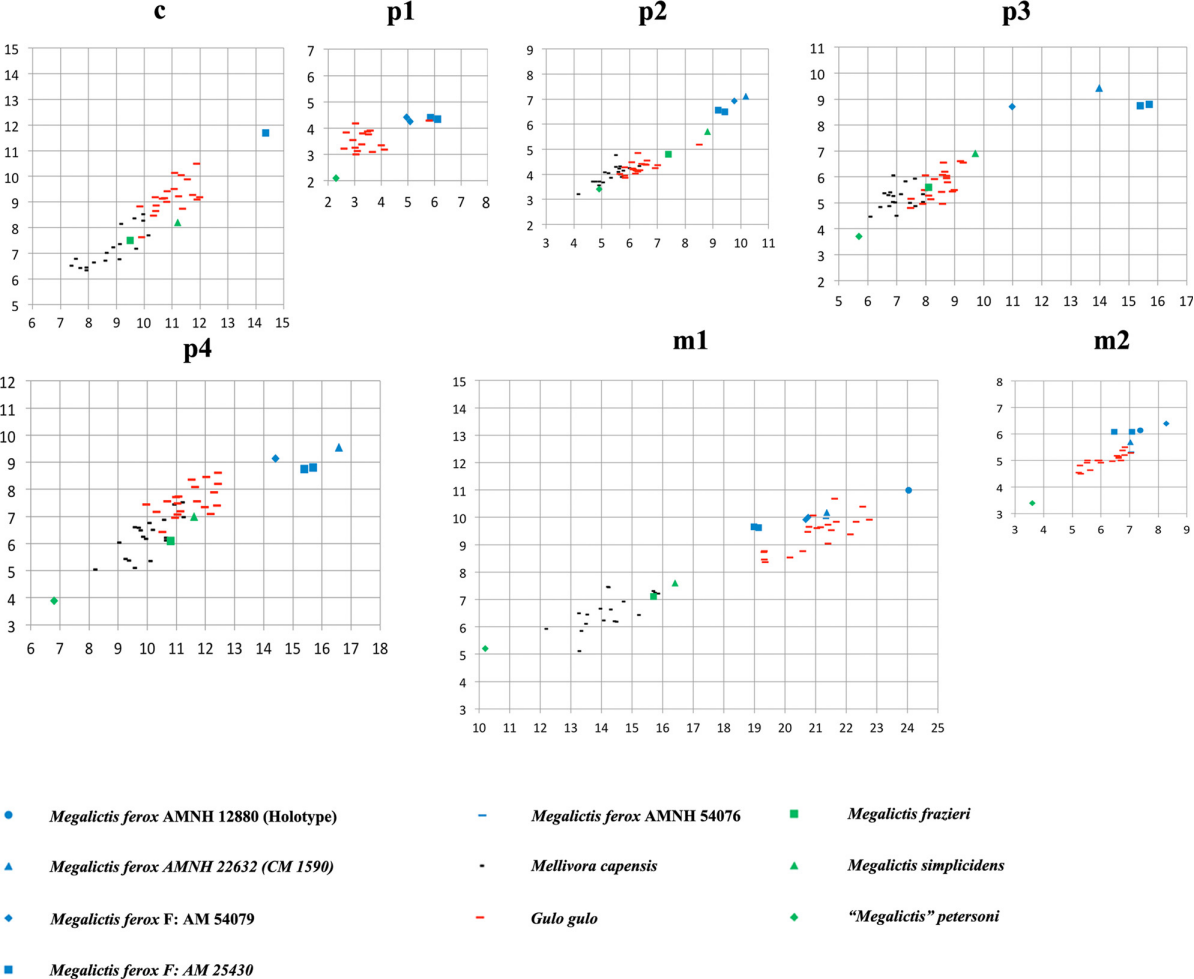
Relationships between lengths (L) and widths (W) of lower dentition in *Megalictis ferox*, *Megalictis simplicidens*, *Megalictis frazieri*, and “*Megalictis*” *petersoni*.

Metrically the new *Megalictis ferox* sample described above (F:AM 54079, F:AM 25430 and AMNH 54076) together with AMNH 12880 and CM 1590 form a single picture of *M*. *ferox* with dental biometric variability similar to the largest extant terrestrial mustelids *Gulo* and *Mellivora* (Figs 7 and 8). However, if *M*. *simplicidens* is considered as a synonym of *M*. *ferox*, this variability exceeds the extant one. Such variability is much more pronounced when all the specimens of *M*. *simplicidens*, *M*. *frazieri* and the small “*M*.*” petersoni* (Figs 7 and 8) are included.

### Phylogenetic relationships of the Oligobuninae

*Megalictis* and the other oligobunines *Oligobunis*, *Brachypsalis* (Cope, 1890) [43], *Promartes* Riggs, 1942 [44] and *Zodiolestes* Riggs, 1942 [44], as well as *Potamotherium* Geoffroy, 1833 [45] and *Plesictis* Pomel 1846 [46] have been referred to as “paleomustelids” (a paraphyletic assemblage of early Miocene taxa) in contrast to the “neomustelids” (modern mustelids plus close fossil relatives). The affinities between the paleomustelids and neomustelids are unresolved [2, 16, 47–49]. The taxonomic position of *Potamotherium* is highly controversial because of its convergences in dentition with otters and in postcranial skeleton with phocids and otters. In the online supplemental information, Rybczynski et al. [50] note that *Potamotherium* is “enigmatic”. It has been classified as a mustelid *s*.*s*. (either inside or outside of Lutrinae) [2, 15–17, 51]; other authors allocated the genus outside the Mustelidae [47, 49, 52]. Wolsan [47] and Sato et al., [49], in a study of the phylogenetic relationships of the extant Musteloidea (clade including Mustelidae, Procyonidae, Ailuridae and Mephitidae), named a new family, Semantoridae, for an extinct group of primitive musteloids including *Mustelavus*, *Potamotherium*, *Semantor* and the oligobunines *Megalictis*, *Promartes*, *Oligobunis* and *Brachypsalis*. They divided the Musteloidea into two sublcades: (1) the Semantoridae, an extinct subclade of stem musteloids and (2) a crown subclade of Musteloidea (with the lineages of the living musteloids). Therefore Wolsan [47] and Sato et al., 2009 [49] consider *Megalictis* and *Oligobunis* as stem musteloids, not mustelids. Within this entire taxonomic framework, we tested whether the oligobunines (*Megalictis* and *Oligobunis*) are stem mustelids or stem musteloids. Because the postcranial skeleton of *Potamotherium* and *Semantor* is highly specialized, adapted to an aquatic or semiaquatic lifestyle [53–55], and in the absence of dental remains of *Semantor*, both taxa are excluded in our cladistic analysis, which is based only in features of the cranium and dentition and whose purpose is to establish the phylogenetic relationships of the oligobunines that possess a postcranial skeleton adapted to a terrestrial lifestyle [2, 7, 44].

Our cladistic analysis shows that the oligobunines *M*. *ferox*, *M*. *simplicidens*, *M*. *frazieri*, “*M*.*” petersoni* and *O*. *crassivultus* are grouped in a monophyletic clade (Fig 5) with high values of Bootstrap and Bremmer Support. The monophyletic status of the Oligobunines was also demonstrated by Finarelli [16] and Wang et al. [15]. Even though the phylogenetic relationships of modern taxa are more complex than the tree topology obtained by us (e.g., [49, 56–59]), the oligobunines show a sister group relationship with the crown clade of Mustelidae sensu Wolsan and Sato [59] (Fig 5). Wolsan & Sato [59] pointed out a formal phylogenetic definition for Mustelidae, as the smallest clade containing *Mustela erminea* and *Taxidea taxus*. However, according the phylogenetic position of the oligobunines obtained by us, we henceforth use the term mustelid as a total clade including to the extant crown clade of mustelids plus the stem mustelid clade of Oligobunines. A similar interpretation of the relationship of this stem mustelids with the living ones, such as the application of the term Mustelidae was obtained by Baskin [2], Wang et al., [15] and Finarelli [16] even though Finarelli determined *Megalictis* as being a sister group of *G*. *gulo* and *Martes americana*.

### Paleobiology of *Megalictis ferox*

The tendency towards gigantism in Mustelidae, the family that includes the smallest modern carnivoran (*Mustela nivalis*), has occurred in different lineages throughout its evolutionary history. For example, *Megalictis*, *Ekorus*, *Enhydriodon*, *Eomellivora*, *Ferinestrix*, and *Plesiogulo* all exceed the size of the wolverine (*G*. *gulo*), the largest extant terrestrial mustelid [1, 13, 32, 35, 37, 60, 61]. We have estimated the basal cranial length of the *M*. *ferox* specimen AMNH-12880 based on the measurements of F:AM 25430 (Fig 4 and Table 3). Comparing the linear measurements of the cranium and mandible of *M*. *ferox* with some extant and extinct carnivorans [13, 32, 33, 35 37, 62] (Table 3), the basicranial length of *M*. *ferox* is similar to that of *Panthera onca* (jaguar) and overlaps with *C*. *lupus*. It is thus the largest mustelid skull ever known, even larger than the Late Miocene giant mustelids, *Ekorus*, *Eomellivora* and *Plesiogulo* (Table 3). The skull is also very wide–its mastoid width approaches that of *Ursus americanus* (Black bear) and exceeds by far that of the largest extant mustelids, the felids *P*. *onca* and *Puma concolor* (cougar) and the extinct mustelids *Eomellivora ursogulo* and *Plesiogulo* (Table 3). The average total mandible length of *M*. *ferox* (Table 3) is the same as that of *P*. *onca* and larger than *Eomellivora piveteaui*, *Ekorus*, and *Plesiogulo crassa*.

**Table 3:** Craniomandibular measures of *Megalictis ferox* and other giant mustelids and extant North American carnivorans.

			Condylobasal length			Mastoid width			Mandible total length		
Taxa		Source	N	Range (F-M)	Average	N	Range (F-M)	Average	N	Range (F-M)	Average
*Megalictis ferox*	Extinct	This manuscript	2	189.5–241.4**	215.4	2	106.1–136.0	121.1	2	139.6–178.3**	159.1
Ekorus ekakeran*	Extinct	This manuscript	1	-	217.6	-	-	-	1	-	143.2
*Plesiogulo monspessulanus*	Extinct	[35]	-	-	-	1	-	108	-	-	-
*Plesiogulo crassa*	Extinct	This manuscript	1	-	209.6	1	-	88	1	-	145.5
*Eomellivora ursogulo*	Extinct	[33]	1	-	191.5	1	-	91	-	-	-
*Eomellivora piveteaui*	Extinct	[32]	1	-	182.5**	-	-	-	2	120.8–134.1	127.6
*Ursus americanus*	Extant	[62]	89	244.8–275.9	260.3	10	122.5–146.49	134.5	36	156.5–176	166.2
*Canis lupus*	Extant	[62]	660	228.5–241.0	234.8	-	-	-	299	175.3–187.4	181.3
*Panthera onca*	Extant	[62]	112	177–276	218.2	7	92.5–103.7	98.1	5	148.3–165.1	159.
*Puma concolor*	Extant	[62]	173	166.4–184.2	175.5	20	68.3–83.8	76.0	75	123.9–141.7	132.8
*Canis latrans*	Extant	[62]	170	166.8–173.6	170.2	101	59.8–60.5	60.7	83	133.7–140.2	136.9
*Gulo gulo*	Extant	[62]	192	133.9–144.9	139.4	8	78.5–90.7	85.4	20	93.6–103.5	98.5
*Enhydra lutris*	Extant	[62]	272	127.7–134.9	131.3	16	91.7–99.7	95.7	16	80–87.4	83.7

*Cast

** Inferred

F = Female

M = Male

For extinct taxa the sex is unknown.

**Fig 9:**
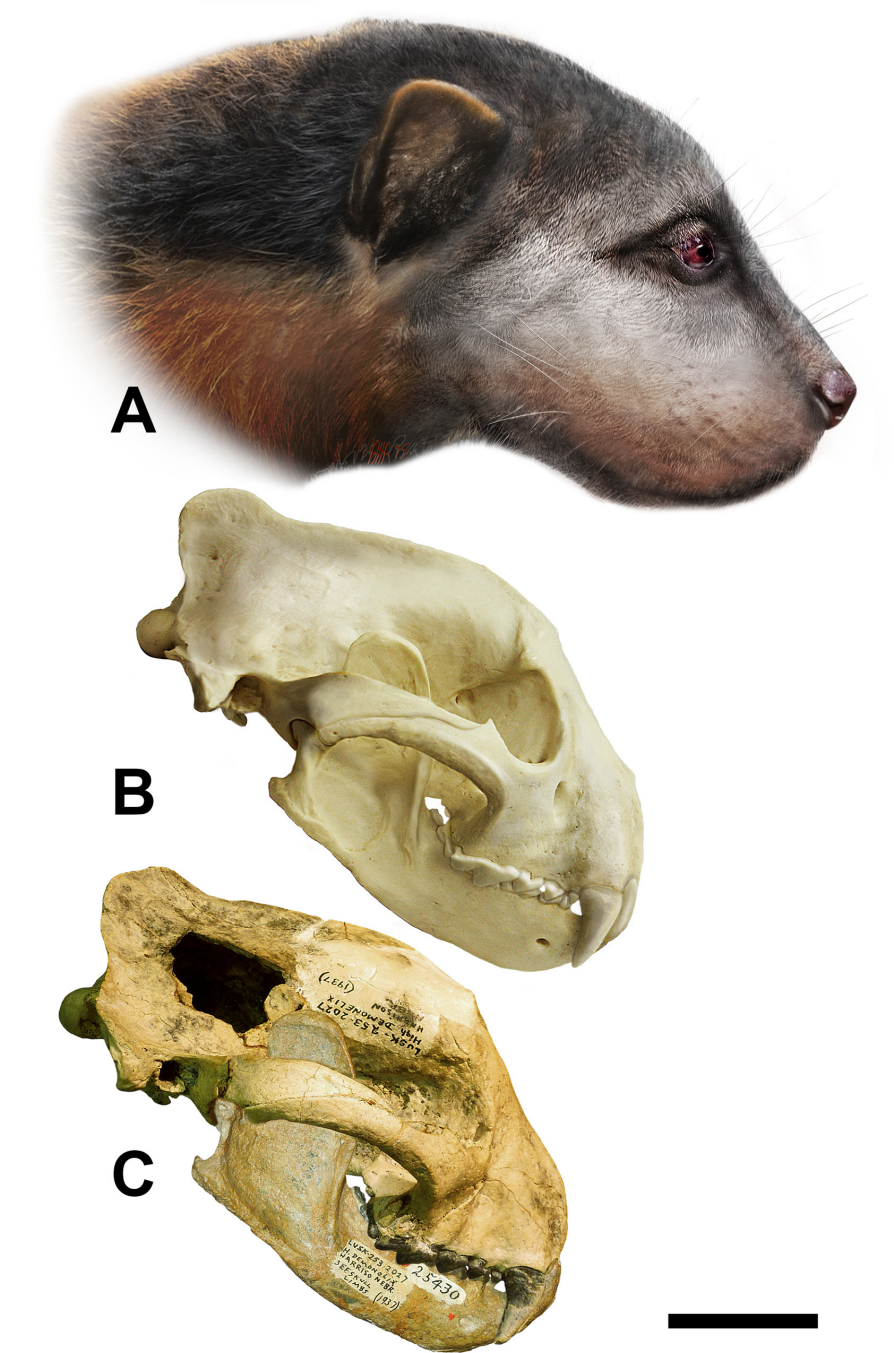
Sequential reconstruction of the head of *Megalictis ferox* based on F:AM 25430. A life appearance; B, reconstructed skull and mandible; C, Skull and mandible F:AM 25430. Artwork by Adam Hartstone-Rose.

Matthew [1] published a reconstruction of the skull and mandibles of *M*. *ferox* AMNH-12880 that, in light of this study of new specimens, was clearly misinterpreted. His reconstruction has an overly-shortened rostrum and a very high forehead–all of which suggest a cat-like morphology (e.g., [7, 63, 64]). The F:AM 25430 specimen of *M*. *ferox* has features that differ from Matthew’s reconstruction in its stouter premolars and molars, longer rostrum, and a smaller forehead. This morphology corresponds to a more bone-crushing hyena-like ecomorphotype, than Matthew’s more hypercarnivorous reconstruction suggests (Fig 9, S6 Video). That is, the dentition of *M*. *ferox* represents that of a durophagous diet, more similar to that of, among extant mustelids, the wolverine [65]. The relatively blunt teeth (low Radius-of-Curvature) and low Intercuspid-Notch scores also support a relatively durophagous diet [66, 67]. The especially enlarged anterior edge of the *Megalictis* coronoid process, where the tendon of the M. *temporalis* is attached, could indicate adaptation for a wider gape. This feature, indicating emphasis on the longer anterior fibers of this muscle, is also present in hyaenids (*Crocuta*, *Hyaena* and *Parahyaena*) and jaguar (*P*. *onca*), all carnivorans with powerful bite forces that eat larger prey [68]. This implies that the *temporalis* anchors more significantly on this anterior-most tendon as opposed to the central tendon or the bony faces of the coronoid process. This would allow the muscle fibers to be longer, thus allowing greater overall stretch of the muscle, which then allow greater overall gape [26]. This would be necessary in animals that eat larger prey, especially if they also have shorter faces (e.g., if the linear gape must be accomplished through radial rotation as opposed to elongation of the mandibles). However, this increase in fiber length comes at the cost of contractile force for a given muscle size–longer fibers have greater stretch but fewer of them can pack into the same volume of muscle thus resulting in a relatively reduced physiological cross-sectional area. Thus the temporal muscle in *Megalictis* appears relatively massive suggesting both great force production and gape abilities.

*Megalictis ferox* shares several similarities with the smaller-sized *Enhydrocyon crassidens* Matthew, 1907 [1], a wolverine-like hesperocyonine canid found in the same formation as *M*. *ferox* AMNH-12880, but in older sediments from the lower Arikareean. Both carnivorans have massive lower premolars, reduced upper molars, and zygomatic arches of similar shape and size. The similarities in morphology could indicate convergence in feeding habits. A hyena-like ecomorphotype also was developed in the North American borophagine canids, such as *Aelurodon* and the highly derived *Borophagus*, but did not appear until the beginning of the Barstovian (Middle Miocene) for *Aelurodon* and the Claredonian (Middle—Late Miocene) for *Borophagus* [69]. Due to the fact that *M*. *ferox* was restricted to the Arikareean, it would have been the best candidate for a hyena-like ecomorph because in general terms, canids of the time (e.g. *Osbornodon* and *Cormocyon*) had not yet evolved the battery of craniodental characteristics for crushing bones. With that said, although *Megalictis* did not have the extreme durophagous specializations of modern hyenas or fossil borophagines, they likely were more durophagous than the felid-like ecomporph to which they have been previously ascribed. The large-sized of *M*. *ferox*, together with a stout rostrum and mandibles, an enlargement of I3, a high cranium, and a raised nasal (Fig 9, S6 Video) suggest that it was one of the more powerful predators of the Lower Miocene (Arikareean 4) of the Great Plain of North America, coexisting with other large carnivorans including the amphicyonid *Adilophontes* and *Daphoenodon* [8] all of which likely consumed medium and large-sizes mammals including camels, horses and oreodonts [3].

## Conclusions

The new specimens of *Megalictis ferox* described here (F:AM 54079, F:AM 25430 and AMNH 54076) give us a broader understanding of the morphology of *M*. *ferox* and lead us to conclude that the holotypes of both *M*. *ferox* (AMNH 12880) and *Aelurocyon brevifacies* (CM 1590) are conspecific and thus the latter should be subsumed into *M*. *ferox*. We argue that there are 3 species ascribed to *Megalictis*: *M*. *ferox*, *M*. *frazieri* and *M*. *simplicidens*. However, the fourth potential congener, *“M”*. *petersoni*, might be best ascribed to a different genus. Our cladistic analysis suggests that *M*. *ferox* is the sister taxon of the clade composed by *M*. *simplicidens*—*M*. *frazieri*. Our phylogenetic hypothesis supports the subfamily Oligobuninae as being a stem mustelid.

The preservation of the of *M*. *ferox* specimen F:AM 25430 represents by far the most complete and best preserved craniomandibular specimen of any giant mustelids. Based on the size of the skull, *M*. *ferox* emerges as the largest terrestrial mustelid ever known–even larger than the extinct Late Miocene giant mustelid *Ekorus*, *Eomellivora*, and *Plesiogulo* [13, 32, 33, 35, 37, 70]. This new material sheds light on a new paleobiological interpretation of *Megalictis* as a hyena-like, bone-crushing mustelid, instead of the cat-like ecomorphotype previously ascribed to the genus.

## Supporting Information

S1 Appendix Character used in this analysis. (DOCX)

S2 Appendix Character matrix in nexus format. (PDF)

S3 Appendix Character matrix in pdf format. (NEX)

S1 Table List of the extant specimens of carnivorans used in this paper. (DOCX)

S1 Video Video of the cranium and mandible of *Megalictis ferox* F:AM 25430. (MOV)

S2 Video Video of the cranium and mandible of *Megalictis ferox* F:AM 54079. (MOV)

S3 Video Video of the cranium and mandible of the Holotype of *Megalictis ferox* AMNH-12880. (MOV)

S4 Video Video of the cranium and mandible *Megalictis ferox* AMNH-22632 (cast of CM 1590). (MOV)

S5 Video Video of the mandible of *Megalictis simplicidens* (CM 1553) and *Megalictis frazieri* UF 23928. (MOV)

S6 Video Video of the reconstructed head of *Megalictis ferox* F:AM 25430. (MOV)

## Abbreviations

ACM: Amherst College Beneski Museum of Natural History, Massachusetts, USA
AHR: Comparative Anatomy Research Collection, University of South Carolina School of Medicine, Columbia, USA
AMNH: American Museum of Natural History, Division of Paleontology and Division of Mammalogy, New York, USA
CM: Carnegie Museum of Natural History, Pittsburgh, USA
F:AM: collection housed in the Frick Collection of the Division of Paleontology, AMNH, New York, USA
LGPUT: Laboratory of Geology and Palaeontology, University of Thessaloniki, Greece
MNCN: Museo Nacional de Ciencias Naturales Madrid, Spain
MNCNCOMP: Comparative Anatomy Research Collection of Paleobiology department of Museo Nacional de Ciencias Naturales Madrid, Spain
NRM: Naturhistoriska rikmuseet, Stockholm, Sweden
PIN: Palaeontological Institute, Russian Academy of Sciences, Moscow, Russia
SAM-PQL: Iziko South African Museum, Cape Town South African Museum, South Africa
TMM: Texas Memorial Museum at the Vertebrate Paleontology Laboratory, the Jackson School of Geosciences, University of Texas, Austin, USA
UF: Vertebrate Paleontology Collection of the Florida Museum of Natural History (FLMNH), University of Florida, Gainesville, USA
UNSM: University of Nebraska State Museum, Lincoln, Nebraska, USA
USNM: United States National Museum of Natural History, Smithsonian Institution, Washington, D.C., USA
