# Supplementary material for: Megalictis, the Bone-Crushing Giant Mustelid (Carnivora, Mustelidae, Oligobuninae) from the Early Miocene of North America
Source: PLoS One. 2016 Apr 7;11(4):e0152430. doi: 10.1371/journal.pone.0152430 (PMC4824437; doi:10.1371/journal.pone.0152430)
Supplement: S1 Appendix — (DOCX) [file pone.0152430.s001.docx]

**Appendix S1. Character state definitions used in this analysis.**

|  |  |  |  |  |
| --- | --- | --- | --- | --- |
|  | Character | Estate | Source | Original character number |
| 1 | Cranial opening of palatine canal | Caudally located, at P4-M1 level (0) | Modified from Bryant et al., 1993 | 1 |
|  |  | Cranially located at P2-3 level (1) |  |  |
| 2 | Alisphenoid canal | Present (0) | Wolsan, 1993 | 5 |
|  |  | Absent (1) |  |  |
| 3 | Incisive foramen | Located at the level of C (0) | This manuscript |  |
|  |  | Located at the level of the diastema I3-C (1) |  |  |
| 4 | Position of the articular process of the mandible in relation to the lower dentition | At the base of the lower dentition (0) | This manuscript |  |
|  |  | Dorsally located (1) |  |  |
| 5 | Coronoid process | High (0) | This manuscript |  |
|  |  | Low (1) |  |  |
| 6 | Orientation of the coronoid process | Straight (0) | Baskin, 1998 |  |
|  |  | Caudally curved (1) |  |  |
| 7 | Rostral ridge of the coronoid process | Slender (0) | This manuscript |  |
|  |  | Robust. A crest extends from the dorsal border of the coronoid process to below the m2, bordering the cranial part of the masseteric fossa (1) |  |  |
| 8 | Relative position of i2 alveolus compared to the i1 and i3 on the mandibular symphysis | i2 alveolus in line with i1 and i3 alveolus (0) | This manuscript |  |
|  |  | I2 alveolus caudally located compared to i1 and i3 alveolus (1) |  |  |
| 9 | Masseteric fossa | Shallow (0) | This manuscript |  |
|  |  | Deep (1) |  |  |
| 10 | Position of the infraorbital canal relative to upper dentition | At the level of the P3 (0) | This manuscript |  |
|  |  | At the level of the P4 (1) |  |  |
| 11 | Position of the border of the palate | Caudal border of palate situated at level of the most caudal upper tooth (0) | Wolsan, 1993 | 3 |
|  |  | Caudal border of palate extended further the most caudal upper tooth (1) |  |  |
| 12 | Caudal width of palate | Wider than the width between canines (0) | Wyss and Flynn, 1993 | 12 |
|  |  | Subequal (1) |  |  |
| 13 | External auditory meatus | Short (0) | Modified of Wolsan, 1993 | 8 |
|  |  | Long (1) |  |  |
| 14 | Suprameatal fossa | Present (0) | This manuscript |  |
|  |  | Absent (1) |  |  |
| 15 | Bulla | Inflated (0) | Baskin, 1998 |  |
|  |  | Flat (1) |  |  |
| 16 | Paroccipital process | Not reduced (0) | Tedford et al., 2009 | 24 |
|  |  | Reduced (1) |  |  |
| 17 | Shape of upper incisor row | Straight (0) | Wesley-hunt, 2005 | 1 |
|  |  | Parabolic (1) |  |  |
| 18 | Size I3 in relation I1-2 | I3 enlarged (0) | Baskin, 1998 |  |
|  |  | I3 similar in size to I1-2 (1) |  |  |
| 19 | Orientation of Canine | Spreaded out laterally, with an arrangement of the tip nonparallel (0) | This manuscript |  |
|  |  | Ventrally directed, with a parallel arrangement of the tip (1) |  |  |
| 20 | P1 | Present (0) | Valenciano et al., 2015 | 1 |
|  |  | Absent (1) |  |  |
| 21 | P2 | Distal accessory cusp present (0) | Valenciano et al., 2015 | 3 |
|  |  | Absent (1) |  |  |
| 22 | P2 occlusal morphology | Subrectangular (0) | Modified of Valenciano et al., 2015 | 4 |
|  |  | Oval (1); Triangular (2) |  |  |
| 23 | P3 occlusal morphology | Subrectangular with an absent or reduced lingual expansion (0) | Modified of Valenciano et al., 2015 | 7 |
|  |  | Triangular with a well-developed lingual expansion (1) |  |  |
| 24 | P3 Robustness ratio [(maximum width/ maximum length) x 100] | Slender P3 (less than 50) (0) | Valenciano et al., 2015 | 8 |
|  |  | Robust P3 (more than 50) (1) |  |  |
| 25 | P3 distal accessory cuspid | Present (0) | Valenciano et al., 2015 | 12 |
|  |  | Absent (1) |  |  |
| 26 | P4 molar-shape like | Absent (0) | This manuscript |  |
|  |  | Present (1) |  |  |
| 27 | P4 Robustness ratio [(maximum width/ maximum length) x 100] and size of the protocone | Slender P4 (less than 60) with slender protocone (0) | Valenciano et al., 2015 | 15 |
|  |  | Robust P4 (more than 60) with robust protocone (1) |  |  |
| 28 | P4. Orientation of the protocone | Protocone in line with the messiobucal corner or surpassing the messiobucal corner of the tooth (0) | Valenciano et al., 2015 | 16 |
|  |  | Protocone distal to the messiobucal corner (1) |  |  |
| 29 | P4 protocone | Low (0) | This manuscript |  |
|  |  | High (1) |  |  |
| 30 | P4 parastyle | Weak (0) | This manuscript |  |
|  |  | Strong (1) |  |  |
| 31 | P4 carnassial notch | Present (0) | Wolsan, 1993 | 15 |
|  |  | Absent (1) |  |  |
| 32 | P4 hypocone | Hypocone notably smaller than protocone or not differentiated (0) | Wolsan, 1993 | 15 |
|  |  | Hypocone and protocone of P4 prominent and subequal in size (1) |  |  |
| 33 | Strong P4 cingulum surrounding the entire tooth | Absent (0) | This manuscript |  |
|  |  | Present (1) |  |  |
| 34 | Relative size between M1 and P4 | M1 larger than or equal in size to P4 (0) | Wolsan, 1993 | 16 |
|  |  | Mi smaller than P4 (1) |  |  |
| 35 | M1 greater width at the level paracone-metacone | Present (0) | This manuscript |  |
|  |  | Absent (1) |  |  |
| 36 | M1. Development of stylar area | Small area (0) | Modified of Valenciano et al., 2015 | 21, 22 |
|  |  | Enlarged area with a swollen cingulum (1) |  |  |
| 37 | M1. Presence and size of the metaconule | Present and large (0) | Modified of Wolsan and Sotnikova, 2013 | 12 |
|  |  | Absent or very small (1) |  |  |
| 38 | M1. Size of the metacone-paracone | Subequal(0) | This manuscript |  |
|  |  | Paracone clearly larger than metacone (1) |  |  |
| 39 | M1 position of the metaconule | Close to protocone (0) | This manuscript |  |
|  |  | Near to metacone (1) |  |  |
| 40 | M1 presence of paraconule | Present (0) | This manuscript |  |
|  |  | Absent (1) |  |  |
| 41 | M1 paraconule shape | Cuspid-like (0) | This manuscript |  |
|  |  | Crest-like (1) |  |  |
| 42 | M1 postprotocrista | Present (0) | This manuscript |  |
|  |  | Absent (1) |  |  |
| 43 | M1. Ocurrence of M1 lingual cingulum | Mesial and distal cingula of M1 not continuous around lingual base of protocone (0) | Wolsan, 1993 | 18 |
|  |  | Mesial and distal cingula of M1 continuous around lingual base of protocone (1) |  |  |
| 44 | M1. Enlarged distolingual area | Absent (0) | This manuscript |  |
|  |  | Present (1) |  |  |
| 45 | M1 hypocone | Present (0) | This manuscript |  |
|  |  | Absent (1) |  |  |
| 46 | M2 | Non reduced, metacone, paracone and protocone diferenciated (0) | This manuscript |  |
|  |  | Reduced or absent (1) |  |  |
| 47 | p1 | Present (0) | Valenciano et al., 2015 | 26 |
|  |  | Absent (1) |  |  |
| 48 | p2 length compared to p3 | Not reduced (0) | This manuscript |  |
|  |  | Reduced (1) |  |  |
| 49 | p2 distal cingulum | Poorly developed (0) | This manuscript |  |
|  |  | Weak but high-crowned (1); stout and high-crowned (2) |  |  |
| 50 | p3 distal accessory cuspid | Well-developed (0) | Valenciano et al., 2015 | 29 |
|  |  | Absent or poorly-developed (1) |  |  |
| 51 | p3 distal cingulum | Poorly developed (0) | This manuscript |  |
|  |  | Weak but high-crowned (1); stout and high-crowned (2) |  |  |
| 52 | p4. Length ratio in relation to m1 [(maximum length p4/ maximum length m1) x 100] | p4 not enlarged (from 50 to 60) (0) | Valenciano et al., 2015 | 31 |
|  |  | p4 relatively enlarged (more than 60) (1) |  |  |
| 53 | p4. Development of the mesial accessory cuspid | Absent or poorly-developed (0) | Valenciano et al., 2015 | 33 |
|  |  | Present with great height development (1) |  |  |
| 54 | p4 distal accessory cuspid | Well-developed (0) | Valenciano et al., 2015 | 34 |
|  |  | Absent or vestigial (1) |  |  |
| 55 | p4 distal cingulum | Poorly developed (0) | This manuscript |  |
|  |  | Weak but high-crowned (1); stout and high-crowned (2) |  |  |
| 56 | m1 robustness ratio [(maximum width/ maximum length) x 100] | Relatively graceful <50 (0) | This manuscript |  |
|  |  | Relatively stout > 50 (1) |  |  |
| 57 | m1 trigonid | m1 trigonid not widened (0) | Baskin, 1998 |  |
|  |  | m1 trigonid widened (1) |  |  |
| 58 | m1. Basal morphology between the paraconid and protoconid | Weak lingual concavity and weak buccal convexity (0) | This manuscript |  |
|  |  | Strong lingual concavity and strong buccal convexity (1) |  |  |
| 59 | m1 metaconid | Present (0) | Valenciano et al., 2015 | 38 |
|  |  | Absent (1) |  |  |
| 60 | m1 metaconid | Low (0) | This manuscript |  |
|  |  | High (1) |  |  |
| 61 | m1. Height of the protoconid in relation to the paraconid | Protoconid higher than paraconid (0) | This manuscript |  |
|  |  | Protoconid similar in height than the paraconid (1) |  |  |
| 62 | m1 trigonid | Short (0) | This manuscript |  |
|  |  | Long (1) |  |  |
| 63 | m1. Relative length of the talonid with respect the total m1 length | Talonid 1/2 or less of the total length (0) | Modified of Valenciano et al., 2015 | 39 |
|  |  | Talonid approximately 1/3 of the total length (1); Equal or less than ¼ of the total length (2) |  |  |
| 64 | m1. Maximum buccolingual width of m1 | Located in the talonid (0) | Valenciano et al., 2015 | 40 |
|  |  | Narrow talonid without the maximum buccolingual width in the talonid (1) |  |  |
| 65 | m1. Height of hypoconid | Low hypoconid (0) | Modified of Valenciano et al., 2015 | 41 |
|  |  | Tall hypoconid (1) |  |  |
| 66 | m1. position of hypoconid | Labially located (0) | Modified of Valenciano et al., 2015 | 42 |
|  |  | Centrally located (1) |  |  |
| 67 | m1. Shape of hypoconid | Cuspid (0) | This manuscript |  |
|  |  | Trenchant (1) |  |  |
| 68 | m1. Lingual morphology between metacristid and entocristid | Lingual morphology open (0) | This manuscript |  |
|  |  | Lingual morphology closed with a lingual rim in the entoconid position (1); occupied by a conulid (2) |  |  |
| 69 | m1 entoconid | Present (0) | This manuscript |  |
|  |  | Absent (1) |  |  |
| 70 | m2 metaconid | Present (0) | Valenciano et al., 2015 | 43 |
|  |  | Absent (1) |  |  |
| 71 | m2 talonid basin | Talonid basin of m2 distinctly longer than trigonid basin (0) | Modified of wolsan, 1993 | 27 |
|  |  | Talonid and trigonid basin of m2 subequal in length (1) |  |  |
| 72 | m2 entoconid | Present (0) | This manuscript |  |
|  |  | Absent (1) |  |  |
| 73 | m3 | Present (0) | This manuscript |  |
|  |  | Absent (1) |  |  |

Bryant HN, Russell AP, Fitch WD. Phylogenetic relationships within the extant Mustelidae (Carnivora): appraisal of the cladistic status of the Simpsonian subfamilies. Zool J Linn Soc. 1993;108: 301–334.

Baskin JA. Procyonidae. In: Janis CM, Scott KM, Jacobs LL, editors. Evolution of Tertiary mammals of North America, Volume 1: terrestrial carnivores, ungulates, and ungulate-like mammals. Cambridge: Cambridge University Press; 1998 pp. 144**–**151.

Wolsan M. Phylogeny and classification of early European Mustelida (Mammalia, Carnivora). Acta Theriol. 1993;38: 345–384.

Wyss AR, Flynn JJ. A phylogenetic analysis and definition of the Carnivora. In: Szalay FS, Novacek MJ, McKenna MC, editors. Mammal Phylogeny: Placentals. New York : Springer; 1993. pp. 32–52.

Tedford RH, Wang X, Taylor BE. Phylogenetic systematics of the North American fossil Caninae (Carnivora: Canidae). Bull Am Mus Nat Hist. 2009;325: 1–218.

Wesley-Hunt GD, Werdelin L. Basicranial morphology and phylogenetic position of the upper Eocene carnivoramorphan Quercygale. Acta Palaeontol. Pol. 2005;50: 837–846.

Valenciano A, Abella J, Sanisidro O, Hartstone-Rose A, Álvarez-Sierra MA, Morales J. Complete description of the skull and mandible of the giant mustelid *Eomellivora piveteaui* Ozansoy, 1965 (Mammalia, Carnivora, Mustelidae) from Batallones (MN10), Late Miocene (Madrid, Spain). J Vert Paleontol. 2015;35 :4, e934570, DOI: 10.1080/02724634.2014.934570.

Wolsan M, Sotnikova M. Systematics, evolution, and biogeography of the Pliocene stem meline badger *Ferinestrix* (Carnivora: Mustelidae). Zool J Linn Soc. 2013;167: 208–226.
