# Supplementary material for: Megalictis, the Bone-Crushing Giant Mustelid (Carnivora, Mustelidae, Oligobuninae) from the Early Miocene of North America
Source: PLoS One. 2016 Apr 7;11(4):e0152430. doi: 10.1371/journal.pone.0152430 (PMC4824437; doi:10.1371/journal.pone.0152430)
Supplement: S1 Table — (DOCX) [file pone.0152430.s004.docx]

| Taxa | Catalog number |
| --- | --- |
| *Bassariscus astutus* | MNCNCOMP-251 |
| *Canis lupus* | MNCN-16150 |
| *Canis lupus* | NRM-20105317 |
| *Canis lupus* | NRM-20115476 |
| *Canis lupus* | NRM-20115478 |
| *Canis lupus* | NRM-20125003 |
| *Eira barbara* | MNCN-3727 |
| *Eira barbara* | NRM-A590033 |
| *Eira barbara* | NRM-A605240 |
| *Eira barbara* | NRM-A607002 |
| *Eira barbara* | NRM-A587135 |
| *Eira barbara* | NRM-A587136 |
| *Eira barbara* | NRM-A583480 |
| *Eira barbara* | NRM-A591501 |
| *Gulo gulo luscus* | AHR-213006 |
| *Gulo gulo luscus* | AHR-213007 |
| *Gulo gulo luscus* | AHR-213008 |
| *Gulo gulo luscus* | AHR-213009 |
| *Gulo gulo luscus* | AHR-213037 |
| *Gulo gulo luscus* | USNM 275160 |
| *Gulo gulo luscus* | USNM 272316 |
| *Gulo gulo luscus* | USNM A06231 |
| *Gulo gulo luscus* | USNM 265649 |
| *Gulo gulo luscus* | USNM 242705 |
| *Gulo gulo gulo* | USNM 108654 |
| *Gulo gulogulo* | USNM 096147 |
| *Gulo gulo gulo* | NRM-A825005 |
| *Gulo gulo gulo* | NRM-A845012 |
| *Gulo gulo gulo* | NRM-20055154 |
| *Gulo gulo gulo* | NRM-A815010 |
| *Gulo gulo gulo* | NRM-A587719 |
| *Gulo gulo gulo* | NRM-A885007 |
| *Gulo gulo gulo* | NRM-A795005 |
| *Gulo gulo gulo* | NRM-A825004 |
| *Martes martes* | MNCN-14672 |
| *Martes martes* | MNCN-14729 |
| *Martes martes* | MNCN-14738 |
| *Martes martes* | MNCN-M21757 |
| *Mellivora capensis* | AMNH 69499 |
| *Mellivora capensis* | AMNH 81831 |
| *Mellivora capensis* | AMNH 160988 |
| *Mellivora capensis* | AMNH 34263 |
| *Mellivora capensis* | AMNH 81848 |
| *Mellivora capensis* | AMNH 34264 |
| *Mellivora capensis indica* | AMNH 83450 |
| *Mellivora capensis cottoni* | AMNH 51952 |
| *Mellivora capensis* | AMNH 81232 |
| *Mellivora capensis* | AMNH 119949 |
| *Mellivora capensis* | AMNH 119622 |
| *Mellivora capensis* | NMR-A582462 |
| *Mellivora capensis* | NMR-A583405 |
| *Mellivora capensis* | NMR-A605023 |
| *Mellivora capensis cottoni* | NMR-A591017 |
| *Mellivora capensis* | NMR-A580357 |
| *Mellivora capensis* | NMR-A584514 |
| *Mellivora capensis cottoni* | NMR-A591015 |
| *Mellivora capensis* | NMR-A580555 |
| *Mellivora capensis* | USNM 270224 |
| *Mellivora capensis* | USNM 302409 |
| *Mephitis mephitis* | AHR 212001 |
| *Mustela putorius* | MNCN-12456 |
| *Mustela putorius* | MNCN-3823 |
| *Mustela putorius* | MNCN-3824 |
| *Mustela putorius* | MNCN-12523 |
| *Mustela putorius* | MNCN-12531 |
| *Pekania pennanti pacifica* | USNM 051270 |
| *Pekania pennanti pacifica* | USNM 171002 |
| *Pekania pennanti columbiana* | USNM A44497 |
| *Pekania pennanti pacifica* | USNM 087081 |
| *Pekania pennanti columbiana* | USNM A44501 |
| *Taxidea taxus* | USNM 110026 |
| *Taxidea taxus* | USNM 096212 |
| *Taxidea taxus* | USNM170262 |
| *Taxidea taxus* | USNM A44050 |
| *Taxidea taxus* | USNM 072234 |
| *Taxidea taxus* | USNM 223290 |
| *Taxidea taxus* | USNM 347884 |
| *Taxidea taxus* | USNM 132825 |
| *Procyon lotor* | NRM-A589597 |
| *Procyon lotor* | NRM-A584025 |
| *Procyon lotor* | NRM-A985739 |
| *Procyon lotor* | NRM-A601266 |
| *Procyon lotor* | NRM-A582046 |
| *Nasua nasua* | NRM-A583348 |
| *Nasua nasua* | NRM-A587112 |
| *Nasua nasua* | NRM-A587109 |
| *Nasua nasua* | NRM-A587102 |
| *Nasua nasua* | NRM-A595075 |
